# Nucleotide Analog ARL67156 as a Lead Structure for the Development of CD39 and Dual CD39/CD73 Ectonucleotidase Inhibitors

**DOI:** 10.3389/fphar.2020.01294

**Published:** 2020-09-08

**Authors:** Laura Schäkel, Constanze C. Schmies, Riham M. Idris, Xihuan Luo, Sang-Yong Lee, Vittoria Lopez, Salahuddin Mirza, The Hung Vu, Julie Pelletier, Jean Sévigny, Vigneshwaran Namasivayam, Christa E. Müller

**Affiliations:** ^1^ PharmaCenter Bonn, Pharmaceutical Institute, Pharmaceutical Sciences Bonn (PSB), Pharmaceutical & Medicinal Chemistry, University of Bonn, Bonn, Germany; ^2^ Centre de Recherche du CHU de Québec – Université Laval, Québec City, QC, Canada; ^3^ Départment de Microbiologie-Infectiologie et d’Immunologie, Faculté de Médecine, Université Laval, Quebec City, QC, Canada

**Keywords:** ARL67156, CD39, CD73, docking, dual-target inhibitors, ecto-5’-nucleotidase, nucleoside triphosphate diphosphohydrolase1 (NTPDase1), nucleotides

## Abstract

Nucleoside triphosphate diphosphohydrolase1 (NTPDase1, CD39) inhibitors have potential as novel drugs for the (immuno)therapy of cancer. They increase the extracellular concentration of immunostimulatory ATP and reduce the formation of AMP, which can be further hydrolyzed by ecto-5’-nucleotidase (CD73) to immunosuppressive, cancer-promoting adenosine. In the present study, we synthesized analogs and derivatives of the standard CD39 inhibitor ARL67156, a nucleotide analog which displays a competitive mechanism of inhibition. Structure-activity relationships were analyzed at the human enzyme with respect to substituents in the *N* ^6^- and C8-position of the adenine core, and modifications of the triphosph(on)ate chain. Capillary electrophoresis coupled to laser-induced fluorescence detection employing a fluorescent-labeled ATP derivative was employed to determine the compounds’ potency. Selected inhibitors were additionally evaluated in an orthogonal, malachite green assay versus the natural substrate ATP. The most potent CD39 inhibitors of the present series were ARL67156 and its derivatives 31 and 33 with *K_i_* values of around 1 µM. Selectivity studies showed that all three nucleotide analogs additionally blocked CD73 acting as dual-target inhibitors. Docking studies provided plausible binding modes to both targets. The present study provides a full characterization of the frequently applied CD39 inhibitor ARL67156, presents structure-activity relationships, and provides a basis for future optimization towards selective CD39 and dual CD39/CD73 inhibitors.

## Introduction

**Figure 1:**
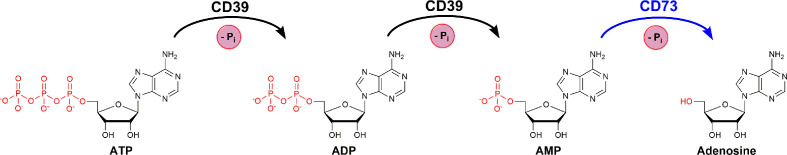
Consecutive hydrolysis of ATP to adenosine by cleaving the terminal phosphate group and releasing inorganic phosphate (P_i_), catalyzed by the enzymes CD39 and CD73.

Nucleoside triphosphate diphosphohydrolase1 (NTPDase1, CD39, EC 3.6.1.5) catalyzes the hydrolysis of extracellular nucleoside tri- and diphosphates producing the corresponding monophosphates (Zimmermann et al., 2012). CD39 is membrane-bound and often co-localized with ecto-5’-nucleotidase (CD73), another ectonucleotidase that further hydrolyzes the nucleoside monophosphates to the corresponding nucleosides (Flögel et al., 2012; Augusto et al., 2013; Bastid et al., 2015). The main substrate of CD39 is ATP which is cleaved *via* ADP to AMP, while AMP acts as the main substrate of CD73 which catalyzes its hydrolysis to adenosine (see **Figure 1**).

Many tumor cells overexpress ectonucleotidases (De Marchi et al., 2019; Horenstein et al., 2019) which metabolize proinflammatory ATP to immunosuppressive, angiogenic, pro-metastatic, and tumor growth-promoting adenosine (Vitiello et al., 2012). Inhibition of CD39 could reduce the production of cancer-promoting adenosine, e.g. in the tumor micro-environment, and increase the concentration of immuno-stimulatory ATP. Due to its pathophysiological role, CD39 represents a promising potential drug target that requires, however, further validation. For this purpose, potent, selective, and metabolically stable inhibitors need to be identified. Besides selective CD39 inhibitors, dual inhibition of CD39 and CD73 is of interest and may be synergistic since the substrate of CD73, extracellular AMP, may additionally be formed by alternative ectonucleotidases, such as nucleotide pyrophosphatase/phosphodiesterase1 (NPP1) (Lee and Müller, 2017; Lee et al., 2017a).

**Figure 2:**
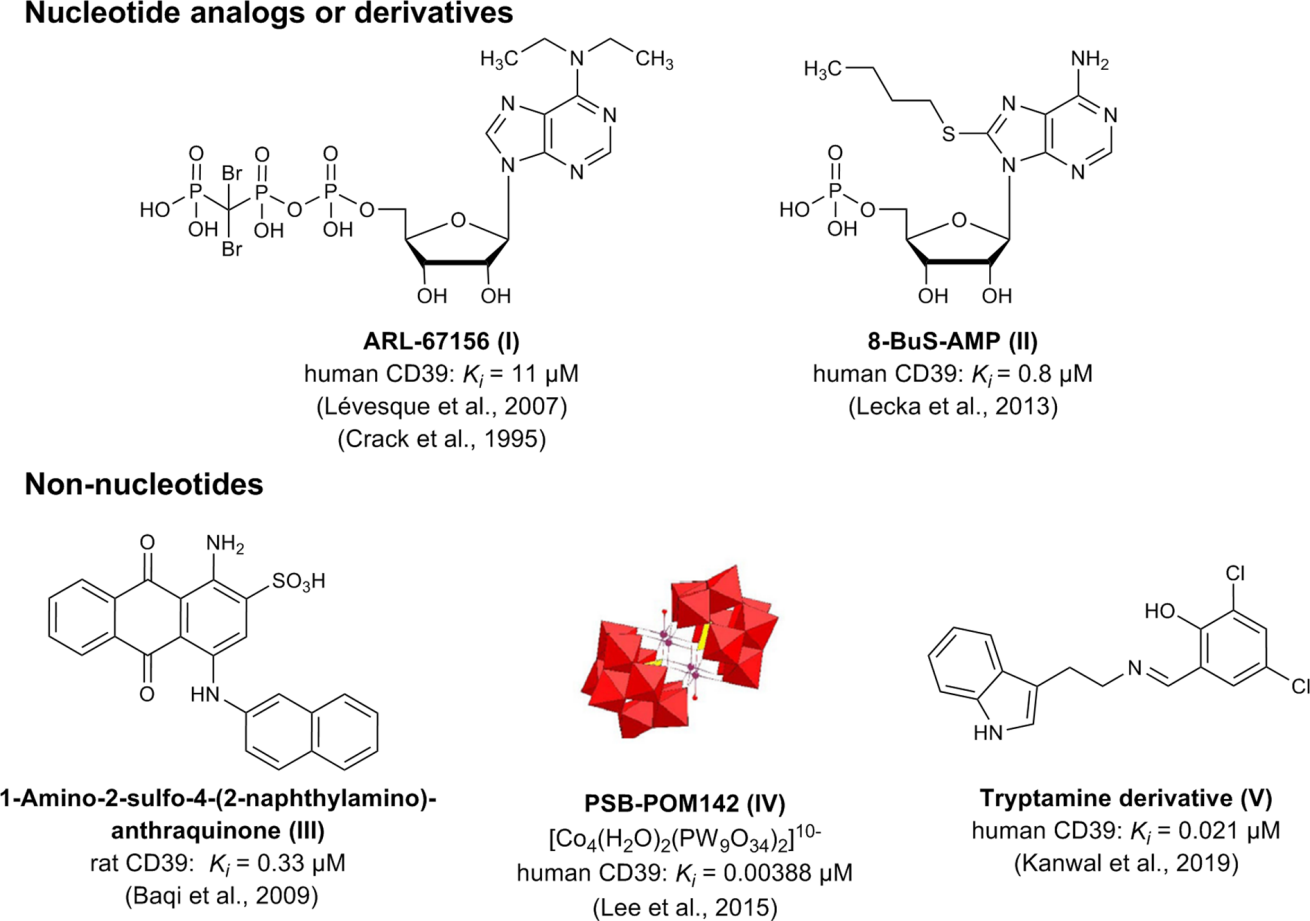
Chemical structures and reported potencies of selected CD39 inhibitors.

Up to now, only moderately potent and/or non-selective CD39 inhibitors are available. These can be divided into (i) nucleotide derivatives and analogs, e.g. *N^6^*-diethyl-β,γ-dibromomethylene-ATP (ARL67156, I) and 8-butylthio-AMP (8-BuS-AMP, II), and (ii) non-nucleotides, including the sulfonate dyes reactive blue 2 (RB-2) and related anthraquinone derivatives (e.g. III), polyoxometalates (e.g. PSB-POM-142, IV), and tryptamine-derived imines (e.g. V) (Crack et al., 1995; Müller et al., 2006; Lévesque et al., 2007; Baqi et al., 2009; Lecka et al., 2013; Lee et al., 2015; Kanwal et al., 2019). A selection of the most potent CD39 inhibitors described so far is depicted in **Figure 2**.

The nucleotide-based competitive CD39 inhibitor *N^6^*-diethyl-β,γ-dibromomethylene-ATP (ARL67156) was developed by Fisons Laboratories (now AstraZeneca, Loughborough, UK) as a probe to study ecto-nucleotidases and purinoceptors (Crack et al., 1995). The nucleotide analog was proposed to be relatively stable towards hydrolysis by ectonucleotidases (CD39; NTPDase2,-3,-8; CD73; NPP1; NPP3) because the cleavage site is blocked by replacement of the β,γ-oxygen atom of the ATP triphosphate chain by a dibromomethylene moiety yielding a phosphonate linkage (Lévesque et al., 2007). ARL67156 (I) was shown to competitively inhibit the mouse and human forms of CD39 [*K_i _*(human) 11 μM], NTPDase3 [*K_i _*(human) 18 μM], and NPP1 [*K_i _*(human) 12 μM], but was reported to have a weaker effect on NTPDase2, NTPDase8, NPP3, and CD73 (Lévesque et al., 2007). Furthermore, in contrast to other NTPDase inhibitors, ARL67156 had no significant effect on P2 receptors due to di-substitution of the exocyclic amino group (Robson et al., 2006). ARL67156 is currently the only commercially available CD39 inhibitor, claimed to be metabolically stable and CD39-selective, and it is therefore frequently used for *in vitro* as well as *in vivo* studies despite its moderate potency (Mandapathil et al., 2010; Zhou et al., 2014; Li et al., 2015). Metabolic stability of ARL67156 has not been sufficiently studied to date, and structure-activity relationships (SARs) are largely unknown.

In this study, we characterized the CD39 inhibitor ARL67156 (I) and used it as a lead structure for studying the SARs of ATP analogs and derivatives as inhibitors of CD39 and other ecto-nucleotidases. Derivatization in the *N^6^*- and 8-position of the adenine ring, as well as replacement of the di-bromomethylene bridge were performed. Selectivity versus a broad range of ecto-nucleotidases and metabolic stability were determined for ARL67156 and selected potent inhibitors. Finally, we performed docking studies to facilitate future drug design efforts.

## Materials and Methods

### Syntheses

#### Materials and Instruments

All reagents were commercially obtained from various producers (Acros, Fluorochem, Merck, Carbosynth, Santa Cruz, Sigma Aldrich, and TCI) and used without further purification, unless otherwise stated. Commercial solvents of reagent grade were used without additional purification or drying. 8-Bromoadenosine was synthesized according to a published procedure (Bhattarai et al., 2015). Reactions were monitored by thin layer chromatography (TLC) using Merck silica gel 60 F254 aluminum sheets and dichloromethane (DCM)/methanol (9:1 or 3:1) as the mobile phase. The TLC plates were analyzed by ultraviolet (UV) light at a wavelength (λ) of 254 nm. Column chromatography was carried out on silica gel 0.040–0.060 mm, pore diameter ca. 6 nm. Anion exchange chromatography was performed on a fast protein liquid chromatography (FPLC) instrument (ÄKTA FPLC, from Amersham Biosciences) with a HiPrep Q Fast Flow sepharose column, 16 x 100 mm (GE Healthcare Life Sciences). Elution of the nucleoside triphosphate analogs was achieved with a linear gradient (5–100%, 0.5 M aqueous ammonium bicarbonate buffer in water, 8 column volumes, flow 1 ml/min). The neutral impurities (e.g. nucleosides) eluted first, followed by charged species (mono-, and finally triphosphate analogs). Semi-preparative high performance liquid chromatography (HPLC) was performed on a Knauer Smartline 1050 HPLC system equipped with a Eurospher-100 C18 column, 250 x 20 mm, particle size 10 μm. The UV absorption was detected at 254 nm. Fractions were collected, and appropriate fractions were pooled, diluted with water, and lyophilized several times, using a CHRIST ALPHA 1-4 LSC freeze dryer, to remove the NH_4_HCO_3_ buffer, yielding the nucleotides as white powders. Mass spectra were recorded on an API 2000 mass spectrometer (Applied Biosystems, Darmstadt, Germany) with a turbo ion spray ion source coupled with an Agilent 1100 HPLC system (Agilent, Böblingen, Germany) using an EC50/2 Nucleodur C18 Gravity 3 μm column (Macherey-Nagel, Düren, Germany), or on a micrOTOF-Q mass spectrometer (Bruker, Köln, Germany) with an ESI-source coupled with an HPLC Dionex Ultimate 3000 (Thermo Scientific, Braunschweig, Germany) using an EC50/2 Nucleodur C18 Gravity 3 μm column (Macherey-Nagel, Düren, Germany). All compounds containing Br atoms (14–16 and 24–38) showed the expected typical isotope distribution pattern (see **Figures S6** and **S7**). UV absorption was detected from 220 to 400 nm using a diode array detector (DAD). Nuclear magnetic resonance (NMR) spectra were recorded on Bruker Avance 500 and Ascend 600 MHz spectrometers. DMSO-d_6_, CD_3_OD, or D_2_O were used as solvents. ^31^P-NMR spectra were recorded at 25°C, and phosphoric acid was used as an external standard. For spectra recorded in D_2_O, 3-(trimethylsilyl)propionic acid sodium salt-d_4_ was used as an external standard. When DMSO-d_6_ was used, spectra were recorded at 30°C. Shifts are given in ppm relative to the external standard (in ^31^P-NMR) or relative to the remaining protons of the deuterated solvent used as internal standard (^1^H-, ^13^C-NMR). Coupling constants are given in Hertz (Hz). The designation used to assign the peaks in the spectra is as follows: singlet (s), doublet (d), triplet (t), quartet (q), multiplet (m), broad (br). Melting points were determined on a Büchi 530 melting point apparatus and are uncorrected.

#### Synthetic Procedures

##### General Procedure for the Synthesis of Compounds 2–7

To 6-chloro-9-(β-D-ribofuranosyl)purine (1, 0.5 g, 1.7 mmol, 1.0 eq) in absolute ethanol (15 ml) the appropriate alkylamine and Et3N (0.1 ml, 1.6 mmol, 0.9 eq) were added. The reaction mixture was refluxed for 6–36 h followed by evaporation of the solvent. Yields for intermediate products **3**–**6** were estimated to be above 70%; however exact yields were not determined because they were used without drying and desalting for the subsequent step; only a small amount was purified for analytical purposes.

##### (2R,3R,4S,5R)-2-(6-(Diethylamino)-9H-purin-9-yl)-5-(hydroxymethyl)tetrahydrofuran-3,4-diol (2)

The compound was synthesized using *N,N*-diethylamine (0.3 ml, 3.4 mmol, 2.0 eq) and purified by silica gel column chromatography (CH_3_OH/DCM 2:23) yielding a white powder (0.50 g, 100%). ^1^H-NMR (500 MHz, DMSO-d_6_) δ 8.34 (s, 1H, NC*H*=N) 8.19 (s, 1H, NC*H*=N) 5.89 (d, 1H, *J* = 6.04 Hz, C*H*N) 5.39 (d, 1H, *J* = 6.19 Hz, CHO*H*) 5.33 (dd, 1H, *J* = 4.59, 7.02 Hz, CH_2_O*H*) 5.13 (d, 1H, *J* = 4.61 Hz, CHO*H*) 4.58 (q 1H, *J* = 6.04 Hz, C*H*OH) 4.14 (td, 1H, *J* = 3.36, 4.82 Hz, C*H*OH) 4.03 [br s, 4H, N(C*H*_2_CH_3_)_2_] 3.95 (q, 1H, *J* = 3.54 Hz, C*H*CH_2_) 3.66–3.54 (d m, 2H, CHC*H*_2_) 1.19 [t, 6H, *J* = 6.95 Hz, N(CH_2_C*H*_3_)_2_]. ^13^C-NMR (125 MHz, DMSO-d_6_) δ 153.27, 151.95, 150.06, 138.96, 119.47,87.94, 85.91, 73.57, 70.70, 61.73, 42.56, 13.48. LC/ESI-MS (m/z): positive mode 324.1 [M+H]^+^. Purity determined by HPLC-UV (254 nm)-ESI-MS: 99.2%. mp: 180°C.

##### (2R,3R,4S,5R)-2-(6-(Dimethylamino)-9H-purin-9-yl)-5-(hydroxymethyl)tetrahydrofuran-3,4-diol (3)

The compound was synthesized using *N,N*-dimethylamine (0.1 ml, 1.75 mmol, 1.0 eq) and purified by silica gel column chromatography (CH_3_OH/DCM 1:49) yielding a white powder (0.52 g). ^1^H-NMR (500 MHz, DMSO-d_6_) δ 8.35 (s, 1H, N=C*H*N) 8.20 (s, 1H, N=C*H*N) 5.90 (d, 1H, *J* = 5.97 Hz, C*H*N) 5.39 (d, 1H, *J* = 6.17 Hz, CHO*H*) 5.32 (dd, 1H, *J* = 4.62, 6.95 Hz, CH_2_O*H*) 5.13 (d, 1H, *J* = 4.78 Hz, CHO*H*) 4.56 (q, 1H, *J* = 5.99 Hz, C*H*OH) 4.14 (m, 1H, C*H*CH_2_) 3.95 (q, 1H, *J* = 3.55 Hz, C*H*OH) 3.66–3.55 (d m, 2H, CHC*H*_2_) 3.45 [br s, 6H, N(C*H*_3_)_2_]. ^13^C-NMR (125 MHz, DMSO-d_6_) δ 154.46, 151.82, 150.05, 138.69, 119.94, 87.94, 85.88, 73.64, 70.65, 61.68, 11.57. LC/ESI-MS (m/z): positive mode 296.0 [M+H]^+^. Purity determined by HPLC-UV (254 nm)-ESIMS: 98%. mp: 186°C (lit. 184°C) (Čechová et al., 2011).

##### (2R,3R,4S,5R)-2-(6-(Ethyl(methyl)amino)-9H-purin-9-yl)-5-(hydroxymethyl)tetrahydrofuran-3,4-diol (4)

The compound was synthesized using *N*-ethylmethylamine (0.2 ml, 1.75 mmol, 1.0 eq) yielding a white powder (0.93 g). ^1^H-NMR (500 MHz, DMSO-d_6_) δ 8.35 (s, 1H, N=C*H*N) 8.20 (s, 1H, N=C*H*N) 5.90 (d, 1H, *J* = 6.00 Hz, C*H*N) 5.39 (d, 1H, *J* = 6.19 Hz, CHO*H*) 5.32 (dd, 1H, *J* = 4.61, 6.96 Hz, CH_2_O*H*) 5.13 (d, 1H, *J* = 4.76 Hz, CHO*H*) 4.57 (q, 1H, *J* =5.99 Hz, C*H*OH) 4.14 (m, 1H, C*H*CH_2_) 4.04 (br s, 2H, NC*H*_2_) 3.95 (q, 1H, *J* = 3.51 Hz, C*H*OH) 3.66–3.54 (d m, 2H, CHC*H*_2_) 3.39 (br s, 3H, NC*H*_3_) 1.17 (t, 3H, *J* = 7.00 Hz, C*H*_3_). ^13^C-NMR (125 MHz, DMSO-d_6_) δ 153.82, 151.89, 150.02, 138.82, 119.69, 87.91, 85.88, 73.59, 70.66, 61.69, 44.78, 35.47, 12.56. LC/ESI-MS (m/z): positive mode 310.0 [M+H]^+^. Purity determined by HPLC-UV (254 nm)-ESI-MS: 98.0%. mp: 101°C.

##### 2R,3S,4R,5R)-2-(Hydroxymethyl)-5-(6-(methyl(propyl)amino)-9H-purin-9-yl)tetrahydrofuran-3,4-diol (5)

The compound was synthesized using *N*-methylpropylamine (0.18 ml, 1.75 mmol, 1.0 eq) and purified by silica gel column chromatography (CH_3_OH/DCM 1:9) yielding a white powder (0.66 g). ^1^H-NMR (500 MHz, DMSO-d_6_) δ 8.35 (s, 1H, N=C*H*N) 8.19 (s, 1H, N=C*H*N) 5.89 (d, 1H, *J* = 5.97 Hz, C*H*N) 5.41 (d, 1H, *J* = 6.16 Hz, CHO*H*) 5.33 (m, 1H, CH_2_O*H*) 5.14 (d, 1H, *J* = 4.64 Hz, CHO*H*) 4.57 (q, 1H, *J* = 5.76 Hz, C*H*OH) 4.14 (d, 1H, *J* =3.62 Hz, C*H*OH) 3.95 (d, 1H, *J* = 3.13 Hz, C*H*CH_2_) 3.66–3.54 (d m, 2H, CHC*H*_2_) 3.16 (br s, 2H, NC*H*_2_) [signals underneath previous peaks: (NCH_3_)] 1.64 (q, 2H, *J* = 7.30 Hz, C*H*_2_) 0.87 (t, 3H, *J* = 7.34 Hz, C*H*_3_). ^13^C-NMR (125 MHz, DMSO-d_6_) δ 154.16, 151.88, 150.10, 138.79, 119.71, 87.92, 85.92, 73.62, 70.71, 61.74, 51.32, 48.75, 21.58, 11.06. LC/ESI-MS (m/z): positive mode 324.1 [M+H]^+^. Purity determined by HPLC-UV (254 nm)-ESI-MS: 97.7%. mp: 178°C.

##### (2R,3R,4S,5R)-2-(6-(Dipropylamino)-9H-purin-9-yl)-5-(hydroxymethyl)tetrahydrofuran-3,4-diol (6)

The compound was synthesized using *N,N*-dipropylamine (0.25 ml, 1.75 mmol, 1.0 eq) and purified by silica gel column chromatography (CH_3_OH/DCM 1:19) yielding a white powder (0.65 g). ^1^H-NMR (500 MHz, DMSO-d_6_) δ 8.35 (s, 1H, N=C*H*N) 8.18 (br s, 1H, N=C*H*N) 5.89 (d, 1H, *J* = 6.05 Hz, C*H*N) 5.40 (d, 1H, *J* = 5.91 Hz, CHO*H*) 5.33 (dd, 1H, *J* = 4.63, 6.97 Hz, CH_2_O*H*) 5.14 (d, 1H, *J* = 4.60 Hz, CHO*H*) 4.58 (q, 1H, *J* = 5.66 Hz, C*H*OH) 4.13 (q, 1H, *J* = 4.53 Hz, C*H*OH) 4.06 [m, 4H, N(C*H*_2_)_2_] 3.95 (q, 1H, *J* = 3.50 Hz, C*H*CH_2_) 3.65–3.54 (d m, 2H, CHC*H*_2_) 1.64 [m, 4H, (C*H*_2_)_2_] 0.89 [t, 6H, J = 7.37 Hz, (C*H*_3_)_2_]. ^13^C-NMR (125 MHz, DMSO-d_6_) δ 153.80, 151.88, 150.10, 138.89, 119.50, 87.92, 85.92, 73.56, 70.73, 61.92, 56.17, 48.74, 18.70, 11.18. LC/ESI-MS (m/z): positive mode 352.1 [M+H]^+^. Purity determined by HPLC-UV (254 nm)-ESI-MS: 98.3%. mp: 145°C.

##### (2R,3R,4S,5R)-2-(6-(Ethyl(propyl)amino)-9H-purin-9-yl)-5-(hydroxymethyl)tetrahydrofuran-3,4-diol (7)

The compound was synthesized using *N*-ethylpropylamine (0.2 ml, 1.75 mmol, 1.0 eq) and purified by silica gel column chromatography (CH_3_OH/DCM 1:9) yielding a white powder (0.38 g, 65%). ^1^H-NMR (500 MHz, CD_3_OD) δ 8.15 (d, 2H, *J* = 2.01 Hz, 2x N=C*H*N) 5.93 (d, 1H, *J* = 6.55 Hz, C*H*N) 4.74 (dd, 1H, *J* = 5.15, 6.48 Hz, C*H*OH) 4.30 (dd, 1H, *J* = 2.45, 5.09 Hz, C*H*CH_2_) 4.16 (q, 1H, *J* = 2.40 Hz, C*H*OH) 3.88–3.72 (d m, 2H, CHC*H*_2_) overlapping with 4.10–3.72 (br s, 4H, 2x NC*H*_2_) 1.73 (m, 2H, CH_2_C*H*_2_CH_3_) 1.25 (t, 3H, *J* = 7.04 Hz, CH_2_C*H*_3_) 0.95 [t, 3H, *J* = 7.39 Hz, (CH_2_)_2_C*H*_3_]. ^13^C-NMR (151 MHz, CD_3_OD) δ 155.40, 152.72, 150.70, 140.17, 121.60, 91.21, 88.17, 75.17, 72.77, 63.58, 51.25, 44.72, 22.52, 13.90, 11.36. LC/ESI-MS (m/z): positive mode 310.0 [M+H]^+^. Purity determined by HPLC-UV (254 nm)-ESI-MS: 97.2%. mp: 160°C.

##### (2R,3R,4S,5R)-2-(6-(Benzylamino)-9H-purin-9-yl)-5-(hydroxymethyl)tetrahydrofuran-3,4-diol (8)

The compound was synthesized according to a published procedure (Shimazaki et al., 1987) and purified by silica gel column chromatography (CH_3_OH/DCM 1:9) yielding a white powder (3.45 g, 96%). ^1^H-NMR (500 MHz, DMSO-*d_6_*) δ: 8.36 (s, 1H, H-8), 8.19 (s, 1H, H-2), 7.33–7.17 (m, 5H, H_arom._), 5.88 (d, *J* = 6.1 Hz, 1H, H-1’), 5.39 (d, *J* = 6.2 Hz, 1H, OH-2’), 5.33 (dd, *J* = 7.1, 4.6 Hz, 1H, OH-5’), 5.13 (d, *J* = 4.7 Hz, 1H, OH-3’), 4.71 [s (br), 2H, N-CH_2_], 4.61 (dd, *J* = 11.3, 6.0 Hz, 1H, H-2’), 4.14 (dd, *J* = 8.2, 4.8 Hz, 1H, H-3’), 3.96 (dd, *J* = 3.5 Hz, 1H, H-4’), 3.68–3.64 (m, 1H, H-5’a), 3.57–3.52 (m, 1H, H-5’b), (1H, NH not visible). ^13^C-NMR (125 MHz, DMSO-*d_6_*) δ: 154.7 (C-6, C_quat._), 152.5 (C-2, CH), 148.6 (C-4, C_quat._), 140.1 (C_arom._, C_quat._), 140.0 (C-8, CH), 128.3 (2 x C_arom._, CH), 127.2 (2 x C_arom._, CH), 126.7 (C_arom._, CH), 119.9 (C-5, C_quat._), 88.1 (C-1’, CH), 86.0 (C-4’, CH), 73.6 (C-2’, CH), 70.8 (C-3’, CH), 61.8 (C-5’, CH_2_), 43.0 (C_benzyl_, CH_2_). LC-ESI-MS (m/z): positive mode 358 [M+H]^+^. Purity determined by HPLC-UV (254 nm)-ESI-MS: 98%. mp: 178–180°C. (Lit. 184–186°C) (Shimazaki et al., 1987).

##### Synthesis of (2R,3S,4R,5R)-2-(Hydroxymethyl)-5-(6-phenethylamino)-9H-purin-9-yl)tetrahydrofuran-3,4-diol (9)

The compound was synthesized according to a published procedure (Shimazaki et al., 1987) and purified by silica gel column chromatography (CH_3_OH/DCM 1:9) yielding a white powder (3.21 g, 86%). ^1^H-NMR (500 MHz, DMSO-*d_6_*) δ: 8.33 (s, 1H, H-8), 8.23 (s, 1H, H-2), 7.87 [s (br), 1H, NH], 7.29–7.16 (m, 5H, H_arom._), 5.88 (d, *J* = 6.1 Hz, 1H, H-1’), 5.40 (d, *J* = 6.2 Hz, 1H, OH-2’), 5.36 (dd, J = 7.2, 4.5 Hz, 1H, OH-5’), 5.14 (d, *J* = 4.6 Hz, 1H, OH-3’), 4.61 (dd, *J* = 6.2, 4.9 Hz, 1H, H-2’), 4.15 (dd, *J* = 4.8, 3.0 Hz, 1H, H-3’), 3.96 (dd, *J* = 3.5 Hz, 1H, H-4’), 3.71 [s (br), 2H, N-CH_2_], 3.69–3.65 (m, 1H, H-5’a), 3.57–3.53 (m, 1H, H-5’b), 2.92 (t, *J* = 9.0 Hz, 2H, CH_2_-Ph). ^13^C-NMR (125 MHz, DMSO-*d_6_*) δ: 154.7 (C-6, C_quat._), 152.5 (C-2, CH), 148.5 (C-4, C_quat._), 139.9 (C-8, CH), 139.6 (C_arom._, C_quat._), 128.8 (2 x C_arom._, CH), 128.4 (2 x C_arom._, CH), 126.2 (C_arom._, CH), 119.9 (C-5, C_quat._), 88.1 (C-1’, CH), 86.0 (C-4’, CH), 73.6 (C-2’, CH), 70.8 (C-3’, CH), 61.8 (C-5’, CH_2_), 41.4 (N-CH_2_), 35.1 (CH_2_-Ph). LC-ESI-MS (m/z): positive mode 372 [M+H]^+^. Purity determined by HPLC-UV (254 nm)-ESI-MS: 96%. mp: 183–185°C. (Lit. 166–168°C) (Shimazaki et al., 1987).

##### Synthesis of (2R,3R,4S,5R)-2-(6-amino-8-(butylthio)-9H-purin-9-yl)-5-(hydroxymethyl)tetrahydro-furan-3,4-diol (12)

To a solution of 8-bromoadenosine (10, 0.5 g, 1.44 mmol, 1.0 eq) in absolute ethanol, thiourea (0.2 g, 2.63 mmol, 1.8 eq) was added. After 7 h of refluxing the solution was allowed to cool down and the resulting precipitate was filtered off. The remaining filtrate was evaporated yielding a yellow oil that was resuspended in a mixture of H_2_O/EtOH 1:1. The solution was adjusted to basic pH with 2 M NaOH. Butyl iodide (0.5 mL, 4.32 mmol, 3.0 eq) was added and the reaction was stirred at rt for 2 h. After extraction with ethylacetate (3 x 100 ml), the organic phase was evaporated. Purification by column chromatography (8% MeOH in DCM) afforded the product as a white solid (0.39 g, 76%) ^1^H-NMR (500 MHz, DMSO-d_6_) δ 8.04 (s, 1H, NC*H*=N) 7.23 (s, 2H, N*H*_2_) 5.77 (d, 1H, *J* = 7.21 Hz, C*H*N) 5.59 (dd, 1H, *J* = 3.47, 8.81 Hz, CHO*H*) 5.36 (d, 1H, *J* = 6.14 Hz, CHO*H*) 5.14 (d, 1H, *J* = 4.54 Hz, CH_2_O*H*) 4.98 (dd, 1H, *J* = 6.14, 11.88 Hz, C*H*CH_2_) 4.16 (m, 1H, C*H*OH) 3.96 (m, 1H, C*H*OH) 3.68–3.50 (d m, 2H, CHC*H*_2_) 3.32–3.27 (d m, 2H overlapping with H_2_O peak, SC*H*_2_) 1.68 (m, 2H, C*H*_2_) 1.41 (m, 2H, C*H*_2_) 0.90 (t, 3H, *J* = 7.27 Hz, CH_2_C*H*_3_). ^13^C-NMR (126 MHz, DMSO-d_6_) δ 184.05, 154.67, 151.39, 150.56, 148.83, 119.74, 89.01, 86.72, 71.40, 71.12, 62.36, 32.22, 31.03, 21.32, 13.56. LC/ESI-MS (m/z): positive mode 356.2 [M+H]^+^. Purity determined by HPLC-UV (254 nm)-ESI-MS: 99.0%. mp: 105°C (lit. 171.5°C) (Halbfinger et al., 1999).

##### Synthesis of (2R,3S,4R,5R)-2-(Hydroxymethyl)-5-(6-(methylamino)-9H-purin-9-yl)tetrahydrofuran-3,4-diol (13)

To 6-chloro-9-(β-D-ribofuranosyl)purine (1, 2.0 g, 7.0 mmol) in absolute ethanol (40 ml), 33 wt % methylamine in absolute ethanol (0.9 ml, 21 mmol, 3 eq) and Et_3_N (2 ml, 14 mmol, 2 eq) were added. After 4 h of refluxing, the solvent was evaporated. Column chromatography (CH_3_OH/DCM 1:9) yielded the product as a white powder (2.0 g, 100%). ^1^H-NMR (500 MHz, DMSO-d_6_) δ 8.32 (s, 1H, NC*H*=N) 8.21 (br s, 1H, NC*H*=N) 7.77 (br s, 1H, N*H*CH_3_) 5.87 (d, 1H, *J* = 6.17 Hz, C*H*N) 5.40 (br s, 1H, CHO*H*) 5.14 (br s, 1H, CHO*H*) 4.59 (t, 1H, *J* = 5.33 Hz, C*H*OH) 4.14 (dd, 1H, *J* = 3.21, 4.75 Hz, C*H*OH) 3.95 (q, 1H, *J* = 3.51 Hz, C*H*CH_2_) 3.66–3.54 (d m, 2H, CHC*H*_2_) 3.05 (m, 3H, NHC*H*_3_). ^13^C-NMR (125 MHz, DMSO-d_6_) δ 156.52, 152.46, 148.22, 139.74, 119.98, 88.05, 86.02, 73.65, 70.77, 61.79, 24.44. LC/ESI-MS (m/z): positive mode 282.3 [M+H]^+^. Purity determined by HPLC-UV (254 nm)-ESI-MS: 99.3%. mp: 132°C (lit. 130-132°C) (Čechová et al., 2011).

##### General Procedure for the Synthesis of 14–16

To a solution of *N^6^*-substituted adenosine (2, 3, or 13, 1.0 eq) in 0.1 M sodium acetate buffer pH 4.0 (15 ml) bromine (5.0 eq) was added. The reaction was stirred at rt overnight and monitored by TLC. The solution was decolorized by the addition of a 40% solution of NaHSO_3_, and the pH of the solution was then adjusted to 7 with 4-N aq. NaOH. The precipitate was filtered off and washed with water.

##### (2R,3R,4S,5R)-2-(8-Bromo-6-(methylamino)-9H-purin-9-yl)-5-(hydroxymethyl)tetrahydrofuran-3,4-diol (14)

The compound was synthesized starting from 13 (1.96 g, 7.0 mmol, 1.0 eq) and afforded a white solid (0.60 g, 25%). ^1^H-NMR (500 MHz, DMSO-d_6_) δ 8.20 (s, 1H, NC*H*=N) 8.02 (s, 1H, N*H*) 5.84 (d, 1H, J =7.08 Hz, C*H*N) 5.45 (q, 1H, *J* = 4.07 Hz, CHO*H*) 5.41 (d, 1H, *J* = 6.77 Hz, CHO*H*) 5.19 (d, 1H, *J* = 4.60 Hz, CH_2_O*H*) 5.07 (dd, 1H, *J* = 6.55, 11.33 Hz, C*H*CH_2_) 4.20 (m, 1H, C*H*OH) 3.97 (dd, 1H, *J* = 4.07, 5.66 Hz, C*H*OH) 3.69–3.49 (d m, 2H, CHC*H*_2_) 2.94 (s, 3H, NHC*H*_3_). ^13^C-NMR (125 MHz, DMSO-d_6_) δ 154.12, 152.58, 149.04, 126.87, 120.40, 90.57, 86.84, 71.34, 70.99, 62.24, 27.10. LC/ESI-MS (m/z): positive mode 346.1 [M+H]^+^. Purity determined by HPLC-UV (254 nm)-ESI-MS: 95.6%. mp: 228°C.

##### (2R,3R,4S,5R)-2-(8-Bromo-6-(dimethylamino)-9H-purin-9-yl)-5-(hydroxymethyl)tetrahydrofuran-3,4-diol (15)

The compound was synthesized starting from 3 (2.0 g, 7.0 mmol, 1.0 eq) and afforded a white solid (0.60 g, 21%). ^1^H-NMR (500 MHz, DMSO-d_6_) δ 8.18 (s, 1H, NC*H*=N) 5.84 (d, 1H, *J* = 6.47 Hz, C*H*N) 5.41 (overlapping q and d, 2H, 2x CHO*H*) 5.19 (d, 1H, *J* = 4.68 Hz, CH_2_O*H*) 5.08 (dd, 1H, *J* = 6.48, 11.80 Hz, C*H*CH_2_) 4.21 (m, 1H, C*H*OH) 3.97 (m, 1H, C*H*OH) 3.70–3.49 (d m, 2H, CHC*H*_2_) 3.41 [br s, 6H, N(C*H*_3_)_2_]. ^13^C-NMR (125 MHz, DMSO-d_6_) δ 153.29, 151.72, 150.88, 126.06, 120.37, 90.68, 86.80, 71.12, 70.96, 62.25, 56.16, 18.68. LC/ESI-MS (m/z): positive mode 374.2 [M+H]^+^. Purity determined by HPLC-UV (254 nm)-ESI-MS: 96.6%. mp: 152°C.

##### (2R,3R,4S,5R)-2-(8-Bromo-6-(diethylamino)-9H-purin-9-yl)-5-(hydroxymethyl)tetrahydrofuran-3,4-diol (16)

The compound was synthesized starting from 2 (1.919 g, 5.9 mmol, 1.0 eq) and afforded a white solid (0.52 g, 23%). ^1^H-NMR (500 MHz, DMSO-d_6_) δ 8.17 (s, 1H, N=CHN) 5.84 (d, 1H, *J* = 6.75 Hz, C*H*N) 5.45 (dd, 1H, *J* = 3.87, 8.57 Hz, CHO*H*) 5.42 (d, 1H, *J* = 5.89 Hz, CHO*H*) 5.20 (d, 1H, *J* = 4.40 Hz, CH_2_O*H*) 5.09 (q, 1H, *J* = 5.92 Hz, C*H*CH_2_) 4.19 (td, 1H, *J* = 2.45, 4.76 Hz, C*H*OH) 3.97 (td, 1H, *J* = 2.97, 4.04 Hz, C*H*OH) 4.19–3.7 [br s, 4H, overlapping with previous peaks N(C*H*_2_CH_3_)_2_] 3.67–3.51 (d m, 2H, CHC*H*_2_) 1.18 [t, 6H, *J* = 6.89 Hz, N(CH_2_C*H*_3_)_2_]. ^13^C-NMR (125 MHz, DMSO-d6) δ 152.14, 151.88, 150.94, 126.35, 119.92, 90.70, 86.85, 71.08, 62.29, 56.19, 42.87, 18.70, 13.65. LC/ESI-MS (m/z): positive mode 402.0 [M+H]^+^. Purity determined by HPLCU-V (254 nm)-ESI-MS: 97.6%.

##### General Procedure for the Synthesis of Compounds 17–20

To the 8-bromo-*N^6^*-substituted adenosine derivatives 14–16 in absolute ethanol (15 ml) the corresponding alkylamine and Et_3_N (0.1 ml, 1.6 mmol, 0.9 eq) were added. The reaction mixture was refluxed for 6–36 h followed by evaporation of the solvent.

##### (2R,3R,4S,5R)-2-(8-(Cyclopropylamino)-6-(methylamino)-9H-purin-9-yl)-5-(hydroxymethyl)tetrahydrofuran-3,4-diol (17)

The compound was synthesized starting from 14 (0.5 g, 1.4 mmol, 1.0 eq), using cyclopropylamine (0.3 ml, 4.2 mmol, 3.0 eq). Purification by column chromatography (CH_3_OH/DCM 1:49) afforded the desired product as a yellow waxy residue (0.18 g, 37%). ^1^H-NMR (500 MHz, DMSO-d_6_) δ 7.98 (s, 1H, N=C*H*N) 7.05 (d, 1H, *J* = 2.63 Hz, N*H*CH_3_) 6.86 (q, 1H, *J* = 4.66 Hz, N*H*CH) 5.87 (d, 1H, *J* = 7.29 Hz, C*H*N) 5.82 (dd, 1H, *J* = 4.35, 6.07 Hz, NHC*H*) 5.15 (d, 1H, *J* = 6.68 Hz, CHO*H*) 5.08 (d, 1H, *J* = 4.35 Hz, CHO*H*) 4.58 (q, 1H, *J* = 6.98, 12.55 Hz, CH_2_O*H*) 4.32 (t, 1H, *J* = 4.96 Hz, C*H*CH_2_) 4.09 (m. 1H, C*H*OH) 3.94 (q, 1H, *J* =2.52 Hz, C*H*OH) 3.61 (m, 2H, CHC*H*_2_) 2.93 (d, 3H, *J* = 4.66 Hz, NHC*H*_3_) 0.66 (m, 2H, C*H*_2_) 0.45 (m, 2H, C*H*_2_). ^13^C-NMR (125 MHz, DMSO-d_6_) δ 152.26, 151.58, 148.87, 137.05, 117.62, 86.49, 85.75, 71.03, 70.84, 61.75, 25.01, 18.67, 6.83, 6.19. LC-MS (m/z): positive mode 337.1 [M+H]^+^. Purity determined by HPLC-UV (254 nm)-ESI-MS: 89.4%. mp: 219°C.

##### (2R,3R,4S,5R)-2-(8-(Butylamino)-6-(methylamino)-9H-purin-9-yl)-5-(hydroxymethyl)tetrahydrofuran-3,4-diol (18)

The compound was synthesized starting from 14 (0.4 g, 1.1 mmol, 1.0 eq) using *N*-butylamine (0.3 ml, 4.2 mmol, 3.0 eq). Purification by column chromatography (CH_3_OH/DCM 1:9) afforded the desired product as a slightly yellow solid (0.36 g, 93%). ^1^H-NMR (500 MHz, DMSO-d_6_) δ 7.95 (s, 1H, N=C*H*N) 6.83 (t, 1H, *J* = 5.51 Hz, N*H*CH_2_) 6.77 (q, 1H, *J* = 4.74 Hz, N*H*CH_3_) 5.89 (d, 1H, *J* = 7.69 Hz, C*H*N) 5.84 (br s, 1H, CH_2_O*H*) 5.19 (br s, 1H, CHO*H*) 5.11 (br s, 1H, CHO*H*) 4.62 (br s, 1H, C*H*CH_2_) 4.11 (br s, 1H, C*H*OH) 3.95 (br d, 1H, *J* = 1.98 Hz, C*H*OH) 3.62 (br s, 2H, CHC*H*_2_) 3.36 (m overlapping with H_2_O, 2H, NHC*H*_2_) 2.92 (d, 3H, *J* = 4.78 Hz, NHC*H*_3_) 1.56 (m, 2H, C*H*_2_) 1.33 (m, 2H, C*H*_2_) 0.89 (t, 3H, *J* = 7.38 Hz, CH_2_C*H*_3_). ^13^C-NMR (125 MHz, DMSO-d_6_) δ 152.01, 151.35, 148.86, 148.59, 117.62, 86.45, 85.78, 71.09, 70.87, 61.79, 42.17, 31.00, 29.44, 27.44, 19.78, 13.19. LC/ESI-MS (m/z): positive mode 353.0 [M+H]^+^. Purity determined by HPLC-UV (254 nm)-ESI-MS: 91.4%. mp: 202°C.

##### (2R,3R,4S,5R)-2-(8-(Butylamino)-6-(dimethylamino)-9H-purin-9-yl)-5-(hydroxymethyl)tetrahydrofuran-3,4-diol (19)

The compound was synthesized starting from 15 (0.5 g, 1.3 mmol, 1.0 eg) using butylamine (0.4 ml, 4.3 mmol, 3.2 eq). Purification by column chromatography (CH_3_OH/DCM 1:24) afforded the desired product as a slightly yellow solid (0.16 g, 33%). ^1^H-NMR (500 MHz, CD_3_OD) δ 8.00 (s, 1H, NCH=N) 6.04 (d, 1H, *J* = 8.08 Hz, C*H*N) 4.76 (dd, 1H, *J* = 5.57, 7.43 Hz, C*H*CH_2_) 4.32 (dd, 1H, *J* = 1.80, 5.60 Hz, C*H*OH) 4.16 (br d, 1H, *J* = 1.80 Hz, C*H*OH) 3.88–3.81 (m, 2H, CHC*H*_2_) 3.47 [s, 6H, N(C*H*_3_)_2_] 2.97 (t, 2H, *J* = 7.47 Hz, NHC*H*_2_) 1.71 (m, 2H, C*H*_2_) 1.46 (m, 2H, C*H*_2_) 1.02 (m, 3H, C*H*_3_). ^13^C-NMR (125 MHz, CD_3_OD) δ 152.09, 150.65, 150.06, 147.41, 118.20, 87.05, 86.16, 71.42, 71.36, 61.69, 42.00, 37.40, 31.16, 19.78, 12.79. LC-MS (m/z): positive mode 235.2, 366.9 [M+H]^+^. Purity determined by HPLC-UV (254 nm)-ESI-MS: 85.9%. mp: 119°C.

##### (2R,3R,4S,5R)-2-(6-(Diethylamino)-8-(methylamino)-9H-purin-9-yl)-5-(hydroxymethyl)tetrahydrofuran-3,4-diol (20)

The compound was synthesized starting from 16 (0.52 g, 1.30 mmol, 1.0 eq) using methylamine (8 M, 33% (w/w) in ethanol, 0.06 ml, 1.31 mmol, 1.0 eq). Purification by column chromatography (CH_3_OH/DCM 2:23) afforded the desired product as a white powder (0.30 g, 67%). ^1^H-NMR (500 MHz, DMSO-d_6_) δ 7.94 (d, 1H, *J* = 0.97 Hz, N=CHN) 6.81 (q, 1H, *J* = 4.38 Hz, N*H*CH_3_) 5.87 (d, 1H, *J* = 7.23 Hz, C*H*N) 5.85 (m, 1H, CH_2_O*H*) 5.17 (d, 1H, *J* = 6.63 Hz, CHO*H*) 5.05 (m, 1H, CHO*H*) 4.65 (q, 1H, *J* = 6.71 Hz, C*H*CH_2_) 4.11 (br s, 1H, C*H*OH) 3.95 (d, 1H, J = 1.96 Hz, C*H*OH) 3.87 [q, 4H, J = 6.09 Hz, N(C*H*_2_)_2_] 3.62 (m, 2H, CHC*H*_2_) 3.08 (q, 3H, *J* = 7.26 Hz, NC*H*_3_) 1.16 [m, 6H, N(CH_2_C*H*_3_)_2_]. ^13^C-NMR (125 MHz, DMSO-d6) δ 151.00, 150.62, 150.51, 148.30, 117.27, 86.55, 85.77, 71.08, 70.81, 61.78, 45.90, 42.07, 28.98,14.04, 8.74. LC/ESI-MS (m/z): positive mode 352.9 [M+H]^+^. Purity determined by HPLC-UV (254 nm)-ESI-MS: 98%. mp: 115°C.

##### Synthesis of (2R,3R,4S,5R)-2-(8-(Butylthio)-6-(methylamino)-9H-purin-9-yl)-5-(hydroxymethyl)tetra-hydrofuran-3,4-diol (21)

To a solution of 14 (0.5 g, 1.4 mmol, 1.0 eq) in absolute ethanol, thiourea (0.2 g, 2.49 mmol, 1.8 eq) was added. After 7 h of refluxing the solution was evaporated yielding a yellow oil that was resuspended in a mixture of H_2_O/EtOH 1:1. The solution was brought to basic pH with 2 M NaOH. 1-Iodobutane (0.5 ml, 4.32 mmol, 3.0 eq) was added and the reaction was stirred at rt for 5 h. After extraction with ethyl acetate (3 x 100 ml), the organic phase was evaporated. Purification by column chromatography (CH_3_OH/DCM 1:24) afforded a white solid. (0.21 g, 42%). ^1^H-NMR (500 MHz, DMSO-d_6_) δ 8.13 (br s, 1H, NC*H*=N) 7.63 (br s, 1H, N*H*CH_3_) 5.77 (d, 1H, *J* = 6.89 Hz, C*H*N) 5.62 (dd, 1H, *J* = 3.61, 8.93 Hz, CH_2_O*H*) 5.37 (d, 1H, *J* = 6.42 Hz, CHO*H*) 5.16 (d, 1H, *J* = 4.29 Hz, CHO*H*) 4.98 (q, 1H, *J* = 6.50 Hz, C*H*CH_2_) 4.15 (m, 1H, C*H*OH) 3.96 (q, 1H, *J* = 3.70 Hz, C*H*OH) 3.68–3.49 (d m, 2H, CHC*H*_2_) 3.26 (m, 2H, SC*H*_2_) 2.96 (br s, 3H, NHC*H*_3_) 1.67 (m, 2H, C*H*_2_) 1.40 (m, 2H, C*H*_2_) 0.89 (t, 3H, *J* = 7.38 Hz, CH_2_C*H*_3_). ^13^C-NMR (125 MHz, DMSO-d_6_) δ 153.80, 151.47, 148.49, 128.29, 127.32, 89.04, 86.79, 71.54, 71.17, 62.41, 32.27, 31.11, 27.17, 21.37, 13.59. LC/ESI-MS (m/z): positive mode 370.1 [M+H]+. Purity determined by HPLC-UV (254 nm)-ESI-MS: 90.1%. mp: 144°C.

##### Synthesis of (2R,3R,4S,5R)-2-(8-(Butylthio)-6-(diethylamino)-9H-purin-9-yl)-5-(hydroxymethyl)tetra-hydrofuran-3,4-diol (22)

Compound 16 (0.74 g, 1.83 mmol, 1.0 eq) was suspended in absolute ethanol (5 ml) and the solution was alkalized with 2 M NaOH. Butanethiol (0.4 ml, 3.7 mmol, 2.0 eq) was added and the reaction mixture was stirred at rt for 5 days. After evaporation, the crude product was subjected to silica gel chromatography. However, separation of starting material and product was not possible. Therefore, the mixture was purified by RP-HPLC (20–100% CH_3_OH in H_2_O in 15 min, 20 ml/min) yielding the desired product as a white powder (0.09 g, 12%). ^1^H-NMR (500 MHz, DMSO-d_6_) δ 8.10 (s, 1H, N=C*H*N) 5.72 (t, 1H, *J* = 6.89 Hz, C*H*N) 5.60 (dd, 1H, *J* = 3.43, 8.71 Hz, CH_2_O*H*) 5.36 (d, 1H, *J* = 5.22 Hz, CHO*H*) 5.16 (m, 1H, CHO*H*) 4.98 (d, 1H, *J* = 5.24 Hz, C*H*CH_2_) 4.15 (s, 1H, C*H*OH) 3.95 (m, 1H, C*H*OH) 4.15–3.65 [large bulb, 4H, underneath other peaks, N(C*H*_2_)_2_] 3.65–3.51 (d m, 2H, CHC*H*_2_) 3.25 (m, 2H, SC*H*_2_) 1.72 (m, 2H, C*H*_2_) 1.40 (m, 2H, C*H*_2_) 1.19 [t, 6H, *J* = 6.69 Hz, N(CH_2_C*H*_3_)_2_] 0.89 [t, 3H, *J* = 7.39 Hz, S(CH_2_)_3_C*H*_3_]. ^13^C-NMR (125 MHz, DMSO-d_6_) δ 151.78, 151.54, 150.81, 147.96, 119.80, 88.99, 86.78, 71.31, 71.16, 62.44, 42.61, 31.88, 31.39, 21.56, 13.60 [missing: N(*C*H_2_CH_3_)_2_]. LC/ESI-MS (m/z): positive mode 412.0 [M+H]^+^. Purity determined by HPLC-UV (254 nm)-ESI-MS: 98.5%. mp: 147°C.

##### Preparation of Triethylammonium Hydrogencarbonate (TEAC) Buffer

A 1 M solution of TEAC was prepared by adding dry ice slowly to a 1 M triethylamine solution in water for several hours until a pH of approximately 7.4−7.6 was indicated using a pH meter.

##### General Procedure for the Synthesis of I and 24–38

Lyophilized adenosine derivatives and proton sponge (1.5 eq) were dissolved in 5 ml of trimethyl phosphate under an argon atmosphere at room temperature. The mixture was cooled to 0°C, and phosphoryl chloride (0.1 ml, 1.3 mmol) was added dropwise. After 5 h of stirring at 0°C, tributylamine (4 eq) and 0.5 M tri-*N*-butylammonium dibromomethylenebisphosphonate solution in DMF (2.5 eq) were added to the mixture simultaneously. After 30 min, a cold 0.5 M aqueous TEAC solution (20 ml, pH 7.4-7.6) was added to the mixture and stirring was continued at room temperature for 1 h. Trimethyl phosphate was extracted with *tert*-butylmethylether (3 x 200 ml) and the aqueous solution was lyophilized. The crude nucleoside triphosphate analogs were purified by fast protein liquid chromatography (FPLC). After equilibration of the column with deionized water, the crude product was dissolved in deionized water and injected into the column. The column was first washed with 5% 0.5 M NH_4_HCO_3_ buffer to remove unbound components. Elution started with a solvent gradient of 5-80% 0.5 M NH_4_HCO_3_ buffer over 8 column volumes followed by an isocratic phase at 80% of 0.5 M NH_4_HCO_3_ buffer. Fractions were collected, and appropriate fractions were pooled and lyophilized several times. The monophosphate and the triphosphate analogs were each purified by preparative HPLC (0–30% acetonitrile in 50 mM NH_4_HCO_3_ buffer within 15 min, 20 ml/min). Fractions were collected and appropriate fractions pooled and lyophilized.

##### (Dibromo((((((2R,3S,4R,5R)-5-(6-(diethylamino)-9H-purin-9-yl)-3,4-dihydroxytetrahydrofuran-2-yl)methoxy)-(hydroxy)-phosphoryl)oxy)(hydroxy)phosphoryl)methyl)-phosphonic Acid (I)

The compound was synthesized starting from 2 (0.32 g, 1.0 mmol, 1.0 eq) affording a white solid (0.03 g, 4%). ^1^H-NMR (500 MHz, D_2_O) δ 8.43 (s, 1H, N=C*H*N) 8.14 (s, 1H, N=C*H*N) 6.11 (d, 1H, *J* = 5.83 Hz, C*H*N) 4.76 (d, 1H, *J* = 5.53 Hz, C*H*OH) 4.63 (m, 1H, C*H*OH) 4.40 (m, 1H, C*H*CH_2_) 4.33 (m, 2H, CHC*H*_2_) 3.85 [br s, 4H, N(C*H*_2_CH_3_)_2_] 1.24 [t, 6H, *J* = 7.07 Hz, N(C*H*_3_)_2_]. ^13^C-NMR (125 MHz, D_2_O) δ 156.09, 155.13, 152.30, 140.63, 121.34, 89.38, 86.70, 77.05, 73.12, 68.09, 57.61, 46.64, 15.47. ^31^P-NMR (202 MHz, D_2_O) δ 7.61 (d, 1P, *J* =13.94 Hz, P_γ_) 0.40 (dd, 1P, *J* = 13.66, 29.09 Hz, P_β_) -10.61 (d, 1P, *J* = 29.33 Hz, P_α_). LC/ESI-MS (m/z): positive mode 719.9052 [M+H]^+^ (calcd. 719.9054), and negative mode 717.8904 [M-H]^-^. Purity determined by HPLC-UV (254 nm)-ESI-MS: 97.5%. mp: 127°C.

##### (Dibromo((((((2R,3S,4R,5R)-5-(6-(dimethylamino)-9H-purin-9-yl)-3,4-dihydroxytetrahydro-furan-2-yl)methoxy)-(hydroxy)phosphoryl)oxy)(hydroxy)phosphoryl)methyl)-phosphonic Acid (24)

The compound was synthesized starting from 3 (0.29 g, 1.0 mmol, 1.0 eq) affording a white solid (0.01 g, 1%). ^1^H-NMR (500 MHz, D_2_O) δ 8.45 (s, 1H, N=C*H*N) 8.17 (s, 1H, N=C*H*N) 6.12 (d, 1H, *J* = 5.92 Hz, C*H*N) 4.78 (m, 1H overlapping with H_2_O, C*H*CH_2_) 4.61 (dd, 1H, *J* = 3.60, 4.99 Hz, C*H*OH) 4.41 (m, 1H, C*H*OH) 4.31 (m, 2H, CHC*H*_2_) 3.42 (br s, 6H, N(C*H*_3_)_2_). ^13^C-NMR (125 MHz, D_2_O) δ 156.66, 154.25, 152.05, 140.97, 121.92, 89.56, 86.89, 77.12, 73.26, 68.16, 51.04, 48.52, 41.92. ^31^P-NMR (202 MHz, D_2_O) δ 7.48 (d, 1P, *J* = 14.23 Hz, P_γ_) -0.73 (dd, 1P, *J* = 14.24, 27.90 Hz, P_β_) -10.65 (d, 1P, *J* = 28.38 Hz, P_α_). LC/ESI-MS (m/z): positive mode 691.8745 [M+H]^+^ (calcd. 691.8742), and negative mode 689.8587 [M-H]^-^. Purity determined by HPLC-UV (254 nm)-ESI-MS: 99.7%. mp: 184°C.

##### (Dibromo((((((2R,3S,4R,5R)-5-(6-(ethyl(methyl)amino)-9H-purin-9-yl)-3,4-dihydroxytetra-hydrofuran-2-yl)methoxy)-(hydroxy)phosphoryl)oxy)(hydroxy)phosphoryl)methyl)-phosphonic Acid (25)

The compound was synthesized starting from 4 (0.3 g, 1.0 mmol, 1.0 eq) affording a white solid (0.08 g, 12%). ^1^H-NMR (500 MHz, D_2_O) δ 8.41 (s, 1H, N=C*H*N) 8.11 (s, 1H, N=C*H*N) 6.10 (d, 1H, *J* = 5.79 Hz, C*H*N) 4.76 (t, 1H, *J* = 4.99 Hz, C*H*OH) 4.61 (t, 1H, *J* = 3.49 Hz, C*H*OH) 4.40 (br s, 1H, C*H*CH_2_) 4.31 (m, 2H, CHC*H*_2_) 3.88 (br s, 2H, NC*H*_2_) 3.30 (br s, 3H, NC*H*_3_) 1.20 (t, 3H, *J* = 7.10 Hz, NCH_2_C*H*_3_). ^13^C-NMR (125 MHz, D_2_O) δ 156.57, 155.00, 152.16, 140.60, 121.57, 89.49, 86.73, 77.08, 73.13, 68.11, 59.78, 48.81, 39.25, 14.75. ^31^P-NMR (202 MHz, D_2_O) δ 7.58 (d, 1P, *J* = 14.50 Hz, P_γ_) 0.22 (q, 1P, *J* = 14.29, 29.14 Hz, P_β_) -10.62 (d, 1P, *J* = 29.27 Hz, P_α_). LC/ESI-MS (m/z): positive mode 705.8896 [M+H]^+^ (calcd. 705.8898), and negative mode 703.8737 [M-H]^-^. Purity determined by HPLC-UV (254 nm)-ESI-MS: 100%. mp: 199°C.

##### (Dibromo((((((2R,3S,4R,5R)-3,4-dihydroxy-5-(6-(methyl-(propyl)amino)-9H-purin-9-yl)tetra-hydrofuran-2-yl)-methoxy)(hydroxy)phosphoryl)oxy)(hydroxy)phosphoryl)-methyl)phosphonic Acid (26)

The compound was synthesized starting from 5 (0.32 g, 1.0 mmol, 1.0 eq) affording a white solid (0.06 g, 9%). ^1^H-NMR (500 MHz, D_2_O) δ 8.43 (s, 1H, N=C*H*N) 8.15 (s, 1H, N=C*H*N) 6.12 (d, 1H, *J* = 5.96 Hz, C*H*N) 4.77 (d, 1H, *J* = 5.58 Hz, C*H*OH) 4.63 (t, 1H, *J* = 4.23 Hz, C*H*OH) 4.41 (br s, 1H, C*H*CH_2_) 4.36–4.24 (d m, 2H, CHC*H*_2_) 3.90 (br s, 2H, NC*H*_2_) 3.55 (br s, 3H, NC*H*_3_) 1.69 (m, 2H, NCH_2_C*H*_2_) 0.89 (t, 3H, *J* = 7.40 Hz, CH_2_C*H*_3_). ^13^C-NMR (125 MHz, D_2_O) δ 157.07, 155.07, 152.33, 140.55, 121.69, 89.60, 86.82, 77.06, 73.22, 68.19, 58.70, 55.17, 39.98, 23.18, 12.99. ^31^PNMR (202 MHz, D_2_O) δ 7.56 (d, 1P, *J* = 13.84 Hz, P_γ_) -0.23 (dd, 1P, *J* = 14.43, 29.03 Hz, P_β_) -10.62 (d, 1P, *J* = 28.61 Hz, P_α_). LC/ESI-MS (m/z): positive mode 719.9050 [M+H]^+^ (calcd. 719.9055), and negative mode 717.8896 [M-H]^-^. Purity determined by HPLC-UV (254 nm)-ESI-MS: 95.6%. mp: 101°C.

##### (Dibromo((((((2R,3S,4R,5R)-5-(6-(dipropylamino)-9H-purin-9-yl)-3,4-dihydroxytetrahydro-furan-2-yl)methoxy)-(hydroxy)phosphoryl)oxy)(hydroxy)phosphoryl)methyl)-phosphonic Acid (27)

The compound was synthesized starting from 6 (0.35 g, 1.0 mmol, 1.0 eq) affording a white solid (0.06 g, 8%). ^1^H-NMR (500 MHz, D_2_O) δ 8.43 (s, 1H, N=C*H*N) 8.15 (s, 1H, N=C*H*N) 6.12 (d, 1H, *J* = 5.88 Hz, C*H*N) 4.76 (d, 1H, *J* = 5.53 Hz, C*H*OH) 4.64 (m, 1H, C*H*OH) 4.40 (m, 1H, C*H*CH_2_) 4.36-4.26 (d m, 2H, CHC*H*_2_) 3.81 [br s, 4H, N(C*H*_2_CH_2_CH_3_)_2_] 1.68 [m, 4H, N(CH_2_C*H*_2_CH_3_)_2_] 0.91 [t, 6H, *J* = 7.40 Hz, N(CH_2_CH_2_C*H*_3_)_2_]. ^13^C-NMR (125 MHz, D_2_O) δ 156.76, 155.12, 152.44, 140.49, 121.54, 89.36, 86.77, 77.06, 73.10, 68.12, 53.51, 50.89, 23.47, 13.12. ^31^P-NMR (202 MHz, D_2_O) δ 7.64 (d, 1P, *J* = 13.87 Hz, P_γ_) 0.78 (q, 1P, *J* = 13.82, 29.45 Hz, P_β_) -10.59 (d, 1P, *J* = 29.59 Hz, P_α_). LC/ESI-MS (m/z): positive mode 747.9349 [M+H]^+^ (calcd. 747.9368), and negative mode 745.9222 [M-H]^-^. Purity determined by HPLC-UV(254 nm)-ESI-MS: 97%. mp: 189°C.

##### (Dibromo((((((2R,3S,4R,5R)-5-(6-(ethyl(propyl)amino)-9H-purin-9-yl)-3,4-dihydroxytetra-hydrofuran-2-yl)methoxy)-(hydroxy)phosphoryl)oxy)(hydroxy)phosphoryl)methyl)-phosphonic Acid (28)

The compound was synthesized starting from 7 (0.33 g, 1.0 mmol, 1.0 eq) affording a white solid (0.05 g, 6%). ^1^H-NMR (500 MHz, D_2_O) δ 8.42 (s, 1H, N=C*H*N) 8.14 (s, 1H, N=C*H*N) 6.10 (d, 1H, *J* = 5.70 Hz, C*H*N) 4.75 (t, 1H, *J* = 5.41 Hz, C*H*OH) 4.63 (m, 1H, C*H*OH) 4.39 (s, 1H, C*H*CH_2_) 4.33 (m, 2H, CHC*H*_2_) 3.78 (br d, 4H, *J* = 56.7 Hz, N(C*H*_2_)_2_) 1.68 (m, 2H, NCH_2_C*H*_2_CH_3_) 1.20 (t, 3H, *J* = 7.05 Hz, C*H*_3_) 0.91 (t, 3 H, *J* =7.39 Hz, C*H*_3_). ^13^C-NMR (125 MHz, D_2_O) δ 156.41, 155.11, 152.37, 140.54, 121.41, 89.36, 86.32, 77.05, 73.16, 68.13, 61.65, 53.08, 47.05, 23.53, 15.39, 13.13. ^31^P-NMR (202 MHz, D2O) δ 7.68 (d, 1P, *J* = 7.68 Hz, P_γ_) 1.10 (dd, 1P, *J* = 13.61, 29.77 Hz, P_β_) -10.59 (d, 1P, *J* = 29.75 Hz, P_α_). LC/ESI-MS (m/z): positive mode 734.1371 [M+H]^+^ (calcd. 734.1373), and negative mode 731.9086 [M-H]^-^. Purity determined by HPLC-UV (254 nm)-ESI-MS: 97.1%. mp: 128°C.

##### (((((((2R,3S,4R,5R)-5-(6-(Benzylamino)-9H-purin-9-yl)-3,4-dihydroxytetrahydrofuran-2-yl)methoxy)(hydroxy)-phosphoryl)oxy)(hydroxy)phosphoryl)dibromomethyl)-phosphonic Acid (29)

The compound was synthesized starting from **8** (0.36 g, 1.0 mmol, 1.0 eq) affording a white solid (0.001 g, recovered from NMR). ^1^H-NMR (600 MHz, D_2_O) δ: 8.52 (s, 1H, H-8), 8.24 (s, 1H, H-2), 7.44–7.33 (m, 5H, H_arom._), 6.15 (d, *J* = 6.6 Hz, 1H, H-1’), 4.84 (s (br), 2H, N-CH_2_), 4.81 (t, *J* = 5.4 Hz, 1H, H-2’), 4.63 (dd, *J* = 5.4, 3.6 Hz, 1H, H-3’), 4.42 (m, 1H, H-4’), 4.36–4.32 (m, 1H, H-5’a), 4.28 – 4.24 (m, 1H, H-5’b), (OHs and NH are not visible). ^13^C-NMR (125 MHz, D_2_O) δ: 157.5 (C-6, C_quat._), 155.7 (C-2, CH), 142.3 (C-8, CH), 141.3 (C-4, C_quat._), 131.6 (2 x C_arom._, CH), 130.2 (C_arom._, CH), 129.7 (2 x C_arom._, CH), 124.7 (C_arom._, C_quat._), 121.8 (C-5, C_quat._), 117.8 (Br-C-Br), 89.5 (C-1’, CH), 87.0 (C-4’, CH), 77.2 (C-2’, CH), 73.3 (C-3’, CH), 68.2 (C-5’, CH_2_), 46.8 (C_benzyl_, CH_2_). ^31^P-NMR (243 MHz, D_2_O) δ: 7.67 (d, *J* = 14.34 Hz, 1P, P*_γ_*), -0.45 (dd, *J* = 14.34, 28.43 Hz, 1P, P*_β_*), -10.52 (d, *J* = 28.43 Hz, 1P, P*_α_*). LC-ESI-MS (m/z): positive mode 753.7 [M+H]^+^. Purity determined by HPLC-UV (254 nm)-ESI-MS: 99.9%.

##### (Dibromo((((((2R,3S,4R,5R)-3,4-dihydroxy-5-(6-(phenethylamino)-9H-purin-9-yl)tetrahydrofuran-2-yl)-methoxy)(hydroxy)phosphoryl)oxy)(hydroxy)phosphoryl)-methyl)phosphonic Acid (30)

The compound was synthesized starting from **9** (0.37 g, 1.0 mmol, 1.0 eq) affording a white solid (0.018 g, 4.8%). ^1^H NMR (600 MHz, D_2_O) δ 8.51 (s, 1H, C8-H), 8.22 (s, 1H, C2-H), 7.28 (s, 5H, aryl), 7.21 (s, 1H, NH), 6.09 (d, J = 5.7 Hz, 1H, C1′-H), 4.59 (t, J = 4.1 Hz, 1H, C3′-H), 4.41 (t, 1H, C4′-H), 4.35–4.28 (m, 2H, C5′-H), 3.87 (s, 2H, CH_2_), 3.01 (s, 2H, CH_2_), ^13^C NMR (151 MHz, D_2_O) δ 143.24 (1C, Cq-aryl), 131.95 (2C, CH-aryl), 131.41 (1C, CH-aryl), 129.48 (1C, CH-aryl), 90.06 (1C, C1′), 86.95 (1C, C2′), 77.35 (1C, C3′), 73.18 (1C, C4′), 68.08 (1C, C5′). ^31^P NMR (243 MHz, D_2_O) δ 7.59 (d, *J* = 14.7 Hz, P_γ_), -0.60 (dd, *J* = 28.7, 14.8 Hz, P_β_), -10.50 (d, *J* = 28.2 Hz, P_α_). LC-ESI-MS (m/z): positive mode 766.9 [M+H]^+^. Purity determined by HPLC-UV (254 nm)-ESI-MS: 99.9%.

##### (((((((2R,3S,4R,5R)-5-(6-Amino-8-(butylthio)-9H-purin-9-yl)-3,4-dihydroxytetrahydrofuran-2-yl)methoxy)(hydroxy)-phosphoryl)oxy)(hydroxy)phosphoryl)dibromomethyl)-phosphonic Acid (31)

The compound was synthesized starting from 12 (0.27 g, 0.76 mmol, 1.0 eq) affording a white solid (0.014 g, 2.5%). ^1^H-NMR (500 MHz, D_2_O) δ 8.17 (s, 1H, N=C*H*N) 6.10 (d, 1H, *J* = 6.23 Hz, C*H*N) 5.19 (t, 1H, *J* = 6.19 Hz, C*H*OH) 4.61 (m, 1H, C*H*OH) 4.39 (dd, 1H, *J* = 6.34, 10.22 Hz, C*H*CH_2_) 4.33 (m, 2H, CHC*H*_2_) 3.29 (m, 2H, SC*H*_2_) 1.73 (m, 2H, C*H*_2_) 1.44 (m, 2H, C*H*_2_) 0.90 (t, 3H, *J* = 7.39 Hz, C*H*_3_). ^13^C-NMR (125 MHz, D_2_O) δ 155.14, 154.91, 153.42, 152.48, 121.74, 90.88, 86.35, 79.70, 72.54, 68.28, 57.53, 35.40, 33.48, 24.09, 15.69. ^31^P-NMR (202 MHz, D_2_O) δ 7.46 (d, 1P, *J* =14.53 Hz, P_γ_) -0.69 (dd, 1P, *J* = 14.69, 29.01 Hz, P_β_) -10.62 (d, 1P, *J* = 28.16 Hz, P_α_). LC/ESI-MS (m/z): positive mode 751.8752 [M+H]^+^ (calcd. 751.8775), and negative mode 749.8619 [M-H]^-^. Purity determined by HPLC-UV (254 nm)-ESI-MS: 100%. mp: 167°C.

##### (Dibromo((((((2R,3S,4R,5R)-5-(8-(cyclopropylamino)-6-(methylamino)-9H-purin-9-yl)-3,4-di-hydroxytetrahydrofuran-2-yl)methoxy)(hydroxy)-phosphoryl)oxy)(hydroxy)phosphoryl)methyl)phosphonic- Acid (32)

The compound was synthesized starting from 17 (0.14 g, 0.41 mmol, 1.0 eq) affording a white solid (7.0 mg, 2%). ^1^H-NMR (500 MHz, D_2_O) δ 8.16 (s, 1H, N=C*H*N) 5.96 (d, 1H, *J* = 7.36 Hz, C*H*N) 4.63 (dd, 1H, *J* = 2.7, 5.7 Hz, C*H*CH_2_) 4.41 (m, 1H, C*H*OH) 4.35 (br s, 1H, C*H*OH) 4.24 (d, 2H, *J* = 11.92 Hz, CHC*H*_2_) 3.10 (s, 3H, NHC*H*_3_) 2.76 (m, 1H, NHC*H*) 0.88 (m, 2H, CHC*H*_2_) 0.8–0.72 (d m, 2H, CHC*H*_2_). ^13^C-NMR (125 MHz, D_2_O) δ 155.34, 152.79, 150.67, 150.23, 117.66, 89.45, 87.11, 73.81, 72.65, 63.36, 50.90, 30.49, 27.18, 9.67. ^31^P-NMR (202 MHz, D2O) δ 7.51 (d, 1P, *J* = 14.60 Hz, P_γ_) -0.84 (dd, 1P, *J* = 14.74, 27.48 Hz, P_β_) -11.16 (d, 1P, *J* = 27.67 Hz, P_α_). LC/ESI-MS (m/z): positive mode 732.8970 [M+H]^+^ (calcd. 732.9007), and negative mode 730.8852 [M-H]^-^. Purity determined by HPLC-UV (254 nm)-ESI-MS: 100%. mp: 232°C.

##### (Dibromo((((((2R,3S,4R,5R)-5-(8-(butylamino)-6-(methylamino)-9H-purin-9-yl)-3,4-dihydroxy-tetrahydrofuran-2-yl)methoxy)(hydroxy)phosphoryl)oxy)-(hydroxy)phosphoryl)methyl)-phosphonic Acid (33)

The compound was synthesized starting from 18 (0.32 g, 1.0 mmol, 1.0 eq) affording a white solid (0.017 g, 2.3%). ^1^H-NMR (500 MHz, D_2_O) δ 8.13 (s, 1H, N=C*H*N) 6.04 (d, 1H, *J* = 7.76 Hz, C*H*N) 4.78 (t, 1H, *J* = 7.82 Hz, C*H*OH) 4.66 (dd, 1H, *J* = 2.16, 5.70 Hz, C*H*OH) 4.45 (m, 1H, 1x CHC*H*_2_) 4.38 (br s, 1H, C*H*CH_2_) 4.24 (m, 1H, 1x CHC*H*_2_) 3.50 (m, 2H, NHC*H*_2_) 3.04 (s, 3H, NHC*H*_3_) 1.67 (m, 2H, C*H*_2_) 1.39 (q, 2H, *J* = 7.48 Hz, C*H*_2_) 0.93 (t, 3H, *J* = 7.40 Hz, C*H*_3_). ^13^C-NMR (125 MHz, D_2_O) δ 154.90, 152.87, 150.47, 150.25, 118.58, 89.15, 87.28, 73.33, 72.84, 68.44, 57.70, 45.31, 33.43, 30.46, 22.31, 16.07. ^31^P-NMR (202 MHz, D_2_O) δ 7.48 (d, 1P, *J* = 16.02 Hz, P_γ_) -0.87 (dd, 1P, *J* = 14.47, 26.89 Hz, P_β_) -11.26 (d, 1P, *J* = 27.48 Hz, P_α_). LC/ESI-MS (m/z): positive mode 748.9324 [M+H]^+^ (calcd. 748.9320), and negative mode 746.9163 [M-H]^-^. Purity determined by HPLC-UV (254 nm)-ESI-MS: 99.0%. mp: 178°C.

##### (Dibromo((((((2R,3S,4R,5R)-5-(8-(butylamino)-6-(dimethylamino)-9H-purin-9-yl)-3,4-di-hydroxytetrahydrofuran-2-yl)methoxy)(hydroxy)-phosphoryl)oxy)(hydroxy)phosphoryl)methyl)-phosphonic Acid (34)

The compound was synthesized starting from 19 (0.1 g, 0.27 mmol, 1.0 eq) affording a white solid (6.0 mg, 1.8%). ^1^H-NMR (500 MHz, D_2_O) δ 8.07 (s, 1H, N=C*H*N) 6.06 (d, 1H, *J* = 7.83 Hz, C*H*N) 4.71 (m, 2H, NC*H*_2_) 4.45 (m, 1H, C*H*OH) 4.38 (br s, 1H, C*H*OH) 4.24 (d, 1H, *J* = 11.78 Hz, C*H*CH_2_) 3.54 (d m, 2H, CHC*H*_2_) 3.42 (s, 6H, N(C*H*_3_)_2_) 1.68 (m, 2H, C*H*_2_) 1.38 (m, 2H, C*H*_2_) 0.93 (t, 3H, *J* = 7.40 Hz, C*H*_3_). ^13^C-NMR (125 MHz, D_2_O) δ 163.50, 154.52, 152.23, 149.19, 119.98, 89.00, 87.24, 73.35, 72.84, 68.47, 56.93, 45.12, 41.61, 33.75, 22.88, 16.03. ^31^P-NMR (202 MHz, D_2_O) δ 6.15 (d, 1P, *J* = 14.67 Hz, P_γ_) -2.22 (dd, 1P, *J* = 14.72, 27.57 Hz, P_β_) -12.61 (d, 1P, *J* = 27.71 Hz, P_α_). LC/ESI-MS (m/z): positive mode 762.9478 [M+H]^+^ (calcd. 762.9477), and negative mode 760.9331 [M+H]^-^. Purity determined by HPLC-UV (254 nm)-ESI-MS: 98%. mp: 193°C.

##### (Dibromo((((((2R,3S,4R,5R)-5-(6-(diethylamino)-8-(methylamino)-9H-purin-9-yl)-3,4-di-hydroxytetrahydrofuran-2-yl)methoxy)(hydroxy)phosphoryl)-oxy)(hydroxy)phosphoryl)methyl)phosphonic Acid (35)

The compound was synthesized starting from 20 (0.08 g, 0.23 mmol, 1.0 eq) affording a white solid (9.0 mg, 4%). ^1^H-NMR (500 MHz, D_2_O) δ 8.04 (s, 1H, N=C*H*N) 6.06 (d, 1H, *J* = 7.82 Hz, C*H*N) 4.72 (m, 1H, C*H*OH) 4.60 (dd, 1H, J = 1.99, 5.68 Hz, CHOH) 4.45 (dd, 1H, *J* = 6.43, 10.55 Hz, C*H*CH_2_) 4.33 (d m, 2H, CHC*H*_2_) 3.88 (m, 4H, N(C*H*_2_CH_3_)_2_) 3.09 (s, 3H, NHC*H*_3_) 1.24 (t, 6H, *J* = 7.06 Hz, N(CH_2_C*H*_3_)_2_). ^13^C-NMR (125 MHz, D_2_O) δ 155.08, 152.57, 151.93, 149.89, 119.72, 89.05, 87.04, 73.36, 72.91, 68.66, 57.89, 46.34, 31.89, 15.61. ^31^P-NMR (202 MHz, D_2_O) δ 7.14 (s, 1P, P_γ_) 0.27 (br s, 1P, P_β_) -10.77 (d, 1P, *J* = 26.2 Hz, P_α_). LC/ESI-MS (m/z): positive mode 748.9295 [M+H]^+^ (calcd. 748.9320), and negative mode 746.9181 [M+H]^-^. Purity determined by HPLC-UV (254 nm)-ESI-MS: 93.7%. mp: 249°C.

##### (Dibromo((((((2R,3S,4R,5R)-5-(8-(butylthio)-6-(methylamino)-9H-purin-9-yl)-3,4-dihydroxy-tetrahydrofuran-2-yl)methoxy)(hydroxy)phosphoryl)oxy)-(hydroxy)phosphoryl)methyl)phosphonic Acid (36)

The compound was synthesized starting from 21 (0.2 g, 0.54 mmol, 1.0 eq) affording a white solid (13.0 mg, 3%). ^1^H-NMR (500 MHz, D_2_O) δ 8.19 (s, 1H, N=C*H*N) 6.11 (d, 1H, *J* = 6.70 Hz, C*H*N) 5.20 (q, 1H, *J* = 6.30 Hz, C*H*OH) 4.62 (dd, 1H, *J* = 4.10, 6.09 Hz, C*H*OH) 4.37 (m, 1 H, C*H*CH_2_) 4.32 (d m, 2H, CHC*H*_2_) 3.26 (m, 2H, SC*H*_2_) 3.08 (s, 3H, NC*H*_3_) 1.71 (m, 2H, C*H*_2_) 1.44 (m, 2H, C*H*_2_) 0.91 (t, 3H, *J* = 7.40 Hz, C*H*_3_). ^13^CNMR (125 MHz, D2O) δ 156.01, 154.39, 153.99, 152.30, 122.21, 90.78, 86.17, 73.53, 72.52, 68.30, 50.37, 35.76, 33.60, 30.30, 24.06, 15.69. ^31^P-NMR (202 MHz, D_2_O) δ 7.49 (d, 1P, *J* = 14.51 Hz, P_γ_) 0.70 (dd, 1P, *J* = 14.28, 27.73 Hz, P_β_) -10.64 (d, 1P, *J* = 28.37 Hz, P_α_). LC/ESI-MS (m/z): positive mode 765.8919 [M+H]^+^ (calcd. 765.8931), and negative mode 763.8787 [M-H]^-^. Purity determined by HPLC-UV (254 nm)-ESIMS: 95.4%. mp: 172°C.

##### (Dibromo((((((2R,3S,4R,5R)-5-(8-(butylthio)-6-(diethylamino)-9H-purin-9-yl)-3,4-dihydroxy-tetrahydrofuran-2-yl)methoxy)(hydroxy)phosphoryl)oxy)-(hydroxy)phosphoryl)methyl)phosphonic Acid (37)

The compound was synthesized starting from 22 (0.1 g, 0.24 mmol, 1.0 eq) affording a white solid (7.0 mg, 4%). ^1^H-NMR (500 MHz, D_2_O) δ 8.18 (s, 1H, N=C*H*N) 6.13 (d, 1H, *J* = 6.41 Hz, C*H*N) 5.16 (t, 1H, *J* = 6.26 Hz, C*H*CH_2_) 4.63 (m, 1H, C*H*OH) 4.38 (dd, 1H, *J* = 4.92, 10.90 Hz, C*H*OH) 4.32 (m, 2H, CHC*H*_2_) 3.92 [br s, 4H, N(C*H*_2_)_2_] 3.30–3.22 (d m, 2H, SC*H*_2_) 1.72 (m, 2H, C*H*_2_) 1.42 (m, 2H, C*H*_2_) 1.26 (t, 6H, *J* = 7.03 Hz, N(CH_2_C*H*_3_)_2_) 0.89 (t, 3H, *J* = 7.39 Hz, C*H*_3_). ^13^C-NMR (125 MHz, D2O) δ 153.66, 153.37, 152.57, 151.90, 122.31, 90.78, 86.33, 73.72, 72.61, 68.29, 50.92, 47.16, 36.19, 34.10, 24.22, 15.75, 15.38. 31P-NMR (202 MHz, D2O) δ 7.48 (d, 1P, *J* = 13.83 Hz, P_γ_) -0.74 (dd, 1P, *J* = 12.88, 25.51 Hz, P_β_) -10.64 (d, 1P, *J* = 28.45 Hz, P_α_). LC/ESI-MS (m/z): positive mode 807.9381 [M+H]^+^ (calcd. 807.9401), and negative mode 805.9304 [M+H]^-^. Purity determined by HPLC-UV (254 nm)-ESI-MS: 92%. mp: 190°C.

##### (((((((2R,3S,4R,5R)-5-(6-amino-9H-purin-9-yl)-3,4-dihydroxytetrahydrofuran-2-yl)methoxy)-(hydroxy)-phosphoryl)oxy)(hydroxy)phosphoryl)dibromomethyl)-phosphonic Acid (38)

The compound was synthesized starting from 23 (0.2 g, 0.75 mmol, 1.0 eq) affording a white powder (0.12 g, 24%). ^1^H-NMR (500 MHz, D_2_O) δ 8.53 (s, 1H, N=C*H*N) 8.25 (s, 1H, N=C*H*N) 6.14 (d, 1H, *J* = 6.0 Hz, C*H*N) 4.79 (s, 1 H, C*H*OH) 4.62 (m, 1H, C*H*OH) 4.41 (m, 1H, C*H*CH_2_) 4.30 (d m, 2H, CHC*H*_2_). ^13^C-NMR (125 MHz, D_2_O) δ 158.49, 155.69, 152.04, 142.81, 121.51, 89.63, 86.95, 77.21, 73.33, 68.20, 57.26. ^31^P-NMR (202 MHz, D_2_O) δ 7.56 (d, 1P, *J* = 14.45 Hz, P_γ_) -0.50 (dd, 1P, *J* = 14.40, 28.55 Hz, P_β_) -10.58 (d, 1P, *J* = 28.56 Hz, P_α_). LC/ESI-MS (m/z): positive mode 663.8407 [M+H]^+^ (calcd. 663.8406), and negative mode 661.8256 [M+H]^-^. Purity determined by HPLC-UV (254 nm)-ESI-MS: 100%. mp: degradation >250°C.

##### Synthesis of (((((((2R,3S,4R,5R)-5-(6-Amino-9H-purin-9-yl)-3,4-dihydroxytetrahydrofuran-2-yl)methoxy)-(hydroxy)-phosphoryl)oxy)(hydroxy)phosphoryl)dichloromethyl)-phosphonic Acid (39)

Adenosine (**23**, 0.2 g, 0.75 mmol, 1.0 eq) and proton sponge (0.24 g, 1.13 mmol, 1.5 eq) were dissolved in 5.0 ml of trimethyl phosphate under an argon atmosphere at room temperature. The mixture was cooled to 0°C and phosphoryl chloride (0.1 ml, 1.3 mmol, 1.7 eq) was added dropwise. After 5 h of stirring at 0°C, tributylamine (4.0 eq) and 0.5 M tri-*N*-butylammonium dichloromethylenebisphosphonate solution in DMF (2.5 eq) were added to the mixture simultaneously. After 30 min, cold 0.5 M aqueous TEAC solution (20 ml, pH 7.4–7.6) was added to the mixture and stirring was continued at room temperature for 1 h. Trimethyl phosphate was extracted with *tert*.-butylmethylether (3 x 200 ml), and the aqueous solution was lyophilized. The crude nucleoside triphosphate analogs were purified by FPLC. After equilibration of the column with deionized water, the crude product was dissolved in deionized water and injected into the column. The column was first washed with 5% 0.5 M NH_4_HCO_3_ buffer to remove unbound components. Elution started with a solvent gradient of 5–80% of 0.5 M NH_4_HCO_3_ buffer over 8 column volumes followed by an isocratic phase of 80% of 0.5 M NH_4_HCO_3_ buffer. Fractions were collected, appropriate fractions were pooled and lyophilized several times. The nucleotide analog was further purified by preparative HPLC (0–30% acetonitrile in 50 mM NH_4_HCO_3_ buffer within 15 min, 20 ml/min). Fractions were collected and appropriate fractions were pooled and lyophilized yielding a white solid (0.05 g, 8%). ^1^H-NMR (500 MHz, D_2_O) δ 8.53 (s, 1H, N=C*H*N) 8.25 (s, 1H, N=C*H*N) 6.14 (d, 1H, *J* = 5.95 Hz, C*H*N) 4.78 (s, 1H, C*H*OH) 4.61 (m, 1H, C*H*OH) 4.41 (br s, 1H, C*H*CH_2_) 4.28 (d m, 2H, CHC*H*_2_). ^13^C-NMR (125 MHz, D_2_O) δ 158.54, 155.74, 152.05, 142.79, 121.52, 89.62, 86.99, 77.21, 73.26, 68.16, 37.53. ^31^P-NMR (202 MHz, D_2_O) δ 7.83 (d, 1P, *J* = 18.36 Hz, P_γ_) 0.16 (dd, 1P, *J* = 18.58, 29.06 Hz, P_β_) -10.55 (d, 1P, *J* = 29.64 Hz, P_α_). LC/ESI-MS (m/z): positive mode 573.9446 [M+H]^+^ (calcd. 573.9445), and negative mode 571.9304 [M+H]^-^. Purity determined by HPLC-UV (254 nm)-ESI-MS: 98.1%. mp: 205°C.

##### Synthesis of (((((((2R,3S,4R,5R)-5-(6-Amino-9H-purin-9-yl)-3,4-dihydroxytetrahydrofuran-2-yl)methoxy)-(hydroxy)-phosphoryl)oxy)(hydroxy)phosphoryl)difluoromethyl)-phosphonic Acid (40)

Adenosine (23, 0.2 g, 0.75 mmol, 1.0 eq) and proton sponge (0.24 g, 1.13 mmol, 1.5 eq) were dissolved in 5.0 ml of trimethyl phosphate under an argon atmosphere at room temperature. The mixture was cooled to 0°C and phosphoryl chloride (0.1 ml, 1.3 mmol, 1.7 eq) was added dropwise. After 5 h of stirring at 0°C, tributylamine (4.0 eq) and 0.5 M tri-*N*-butylammonium difluoromethylenebisphosphonate solution in DMF (2.5 eq) were added to the mixture simultaneously. After 30 min, cold 0.5 M aqueous TEAC solution (20 ml, pH 7.4 - 7.6) was added to the mixture and stirring was continued at room temperature for one hour. Trimethyl phosphate was extracted with *tert*.-butylmethylether (3 x 200 ml) and the aqueous solution was lyophilized. The crude nucleoside triphosphate analog was purified by FPLC. After equilibration of the column with deionized water, the crude product was dissolved in deionized water and injected into the column. The column was washed with 5% 0.5 M NH_4_HCO_3_ buffer to remove unbound components. Elution started with a solvent gradient of 5–80% of 0.5 M NH_4_HCO_3_ buffer over 8 column volumes followed by an isocratic phase of 80% of 0.5M NH_4_HCO_3_ buffer. Fractions were collected, appropriate fractions were pooled and lyophilized several times. The product was further purified by preparative HPLC (0–30% acetonitrile in 50 mM NH_4_HCO_3_ buffer within 15 min, 20 ml/min). Fractions were collected and appropriate fractions pooled and lyophilized yielding a white solid (0.025 g, 6%). ^1^H-NMR (500 MHz, D_2_O) δ 8.52 (s, 1H, N=C*H*N) 8.25 (s, 1H, N=C*H*N) 6.14 (d, 1H, *J* = 6.02 Hz, C*H*N) 4.78 (d, 1H, *J* = 5.60 Hz, C*H*CH_2_) 4.57 (m, 1H, C*H*OH) 4.41 (br s, 1H, C*H*OH) 4.25 (d m, 2H, CHC*H*_2_). ^13^C-NMR (125 MHz, D_2_O) δ 158.39, 155.55, 152.01, 142.77, 121.48, 89.58, 86.87, 71.17, 73.24, 68.07. ^31^P-NMR (202 MHz, D_2_O) δ 3.40 (td, 1P, *J* = 58.87, 79.05 Hz, P_γ_) -4.56 (tdd, 1P, *J* = 28.07, 56.21, 84.20 Hz, P_β_) -10.68 (d, 1P, *J* = 30.49 Hz, P_α_). ^19^F-NMR (202 MHz, D_2_O) δ -19.76 (t, 2F, *J* = 82.12 Hz). LC/ESI-MS (m/z): positive mode 542.0017 [M+H]^+^ (calcd. 542.0049), and negative mode 539.9888 [M+H]^-^. Purity determined by HPLC-UV (254 nm)-ESI-MS: 100%. mp: >231°C (decomposition).

### Biological Assays

#### Chemicals and Materials

ATP, calcium chloride, magnesium chloride, 4-(2-hydroxyethyl)-piperazine-1-ethanesulfonic acid (HEPES), ammonium heptamolybdate, dimethyl sulfoxide (DMSO), malachite green, α,β-methylene-ATP (**41**), α,β-methylene-ADP (**42**), β,γ-methylene-ATP (**43**), and polyvinylalcohol were obtained from Sigma (Steinheim, Germany). Disodium hydrogenphosphate and sulfuric acid were purchased from Carl Roth (Karlsruhe, Germany). *N^6^*-[6-(Fluoresceinyl-5′-carboxamido)hexyl]-ATP (PSB-170621A) was obtained from Jena Bioscience (Jena, Germany). The polyacrylamide-coated capillary [30 cm (10 cm effective length) × 50 µm (id), × 360 µm (od)] was purchased from Chromatographie Service GmbH (Langerwehe, Germany).

#### Expression of the Enzymes

The cDNAs of the human enzymes NPP1, NPP3, NPP5, CD38 and CD73 (Genbank accession no. NM_006258, NM_005021, NM_021572, NM_ 001775, and NM_002526, respectively) were obtained from Origene (Rockville, USA). Soluble enzymes were produced as previously reported with some modifications (Lee et al., 2015; Junker et al., 2019). Briefly, the catalytic domains of the enzymes were amplified and sub-cloned into the expression vector *p*ACGP67 A/B modified with the addition of 9 x histidine tag (His-tag) at the C-terminus (except for NPP1). The plasmids were transfected in Sf9 insect cells using Cellfectin™ II Reagent (Thermo Fisher Scientific, MA, USA) and ProEasy™ baculovirus linearized DNA (Cat.#A10S, AB Vector, LLC). Protein expression was conducted for 48 h at 27°C. The signal peptide sequence of the expression vector shuttled the proteins into the supernatant. The supernatant medium was collected, and the enzymes were purified using HisPur™ Ni^2+^-NTA spin columns according to the manufacturer’s protocol. The protein concentration was determined by the method previously described by Lowry et al. (1951).

#### Human CD39 Preparation

Human umbilical cords were obtained under approved institutional review board protocol (Comité d’Éthique de la Recherche du CHU de Québec – Université Laval) following written consent as previously described (Sévigny et al., 1997). They were minced and homogenized with a polytron in 95 mM NaCl, 0.1 mM phenylmethylsulfonyl fluoride (PMSF), and 45 mM Tris solution, pH 7.6. The homogenates were then filtered through a cheese cloth, centrifuged for 15 min at 600 g, and the supernatants were subsequently centrifuged for 1 h at 100,000 g. The pellets were resuspended in 5 mM Tris buffer solution, pH 8.0 and 10% glycerol. All purification steps were performed at 4°C. The preparations were kept at −80°C.

#### Fluorescence Capillary Electrophoresis Assay for CD39

The enzyme activity assay was performed as previously described (Lee et al., 2018). For inhibition screening, three independent experiments were performed. The concentration of the fluorescent substrate PSB-017621A was 0.5 µM (*K_m_* = 19.6 µM); the assay is highly sensitive and therefore allows the use of low substrate concentrations below the *K*_m_ value which facilitates the identification and characterization of moderately potent competitive inhibitors. Test compounds were initially investigated at a concentration of 10 µM, and 40 ng protein from human umbilical cord membrane preparations containing CD39 were added to initiate the reaction. The reaction buffer contained 10 mM HEPES, 2 mM CaCl_2_, 1 mM MgCl_2_, pH 7.4. The samples were incubated at 37°C for 4 min, and the enzymatic reaction was terminated by heating at 90°C for 5 min. The solution was then diluted 1:20 with reaction buffer to perform separation of nucleotides by capillary electrophoresis (CE) followed by laser-induced fluorescence (LIF) detection. For compounds showing ≥70% inhibition of enzymatic activity, compared to the positive control without inhibitor, concentration-inhibition curves were generated at concentrations ranging from 0.01 to 300 µM. Three independent experiments were performed, and curves were calculated by GraphPad Prism 8 software (GraphPad software, San Diego, CA, USA).

Analysis was carried out using a P/ACE MDQ capillary electrophoresis system (Beckman Instruments, Fullerton, CA, USA). The separation was performed in a *polyacrylamide*-coated capillary [30 cm (10 cm effective length) × 50 µm (id), × 360 µm (od)]. Before each run, the capillary was rinsed with the background electrolyte [50 mM phosphate buffer (pH 6.5)] for 1 min at 30 psi. Samples were electrokinetically injected by applying a voltage of -6 kV for 30 s at the capillary outlet, and the fluorescent nucleotide derivatives were separated by voltage application of -15 kV. Detection was performed at an excitation wavelength of 488 nm and an emission wavelength of 520 nm. Data collection and peak area analysis were performed by the P/ACE MDQ software 32 KARAT obtained from Beckman Coulter (Fullerton, CA, USA).

#### Malachite Green Assay for CD39 and NTPDases2, -3, and -8

The enzymatic activity assay was determined essentially as previously described (Cogan et al., 1999) with a few adaptations. The reaction buffer contained 10 mM HEPES, 2 mM CaCl_2_, 1 mM MgCl_2_, pH 7.4 in a final volume of 50 μl in transparent 96-well half area plates. For CD39 (NTPDase1), we made use of human umbilical cord membranes preparations which express high levels of the enzyme. For the other human NTPDase isoenzymes, we had to resort to recombinant expression. Human umbilical cord membrane preparations (250 ng) natively expressing high amounts of CD39, or the respective recombinant COS-7 cell membrane preparations expressing the appropriate NTPDase isoenzyme (ca. 100 ng of protein depending on enzyme activity) (Sévigny et al., 1997; Lecka et al., 2013) with or without inhibitor were preincubated at 37°C and gentle shaking (Eppendorf Thermomixer comfort at 500 rpm) for 5 min. The amount of enzyme preparation was adjusted to ensure 10–20% of substrate conversion. The reaction was initiated by the addition of 50 µM ATP [*K_m_* (CD39) = 17 µM; *K_m_* (NTPDase2) = 70 µM; *K_m_* (NTPDase3) = 75 µM; *K_m_* (NTPDase8) = 46 µM] (Kukulski et al., 2005). After 15 min of incubation at 37°C with gentle shaking, the reaction was stopped by adding the detection reagents (20 µl malachite green solution, 0.6 mM, and 30 µl ammonium molybdate solution, 20 mM, in sulfuric acid, 1.5 M). The released (inorganic) phosphate was quantified after 20 min of gentle shaking at 25°C by measuring the absorption of the malachite green-phosphomolybdate complex at 600 nm using a BMG PheraStar FS plate reader (BMG Labtech GmbH, Ortenberg, Germany). The corrected absorption was calculated by subtracting the absorption of the negative control samples, which were incubated with denatured enzyme (90°C, 15 min), and the inhibition was calculated as follows:

$$\%\;Inhibition=\frac{\left(B-T\right)}{B}*100\%$$

where B is the average corrected absorption of the positive control without inhibitor and T the corrected absorption in the presence of test compound.

Full concentration-inhibition curves were determined with inhibitor concentrations ranging from 0.1 to 300 µM in the presence of 2% DMSO. Three independent experiments were performed (n = 3) and curves were calculated by the GraphPad Prism 8 software. The *K_i_* value was calculated using the Cheng-Prusoff equation for competitive inhibitors:

$$K_{i}=\frac{IC_{50}}{1+\frac{\left[S\right]}{K_{m}}}$$

#### CD73 Assay

The assay was performed as previously described (Freundlieb et al., 2014). Briefly, it contained with 0.09 µg/ml of soluble human CD73 recombinantly expressed in Sf9 insect cells as described (Junker et al., 2019), the respective test compound, and 5.0 µM [2,8-^3^H]AMP (specific activity 7.4 x 108 Bq/mmol, 20 mCi/mmol) as radioactive substrate in assay buffer consisting of 25 mM Tris buffer, 140 mM NaCl, 25 mM NaH_2_PO_4_ pH 7.4. The enzymatic reaction was performed for 25 min at 37°C in a shaking water bath. Then, 500 µl of cold precipitation buffer (100 mM LaCl_3_, 100 mM sodium acetate, pH 4.0) were added to precipitate free phosphate and unconverted [2,8-^3^H]AMP. After 30 min on ice, filtration through GF/B glass fiber filters using a cell harvester was used to separate AMP from adenosine. After washing each reaction vial three times with 400 µl of cold (4**°**C) demineralized water, aliquots of the filtrate were taken, and 5 ml of scintillation cocktail (ULTIMA Gold XR9) was added. The amount of formed adenosine was quantified by liquid scintillation counting (TRICARB 2900 TR, Packard/PerkinElmer).

#### NPP1 Assay

Inhibition of NPP1 was determined as previously described (Lee et al., 2017b). *p*-Nitrophenyl-5’-thymidine monophosphate (*p*-Nph-5’-TMP) was used as an artificial substrate which results in the formation of the *p*-nitrophenolate anion with an absorption maximum of 400 nm. Purified soluble NPP1 [0.36 µg, expressed in Sf9 insect cells as previously described (Lee et al., 2015)] was mixed with test compound (20 µM final concentration for initial screening, 0.1–200 µM for determining concentration-dependent inhibition curves), 2% DMSO and 400 µM of *p*-Nph-5’-TMP as a substrate in a final volume of 100 µl. The mixture was incubated for 30 min at 37°C with gentle shaking, and the enzyme reaction was terminated by the addition of 20 µl of 1 M NaOH. The absorption was measured at 405 nm using a BMG PheraStar FS plate reader (BMG Labtech GmbH, Ortenberg, Germany).

#### NPP4 Assay

Soluble NPP4 was expressed in Sf9 insect cells as recently described in detail (Lopez et al., 2020). Diadenosine tetraphosphate (AP_4_A) was employed as a substrate which is cleaved by NPP4 to ATP and AMP. The reaction product ATP was quantified by luciferin-luciferase reaction (Lopez et al., 2020). A mixture of 1.4 µg** **of NPP4 (soluble form expressed in insect cells and purified) (Lopez et al., 2020), 10 µM of test compound, 2 % DMSO, and 20 µM of AP_4_A as a substrate were incubated for 60 min at 37°C with gentle shaking. The reaction was terminated by heating at 90°C for 5 min, and after cooling down on ice, 50 µl of D-luciferin dissolved in buffer (300 mM Tris-HCl, 15 mM MgCl_2_, 100 ng D-luciferin, pH 7.8) and 50 µl of luciferase (50 ng dissolved in H_2_O) were added. The firefly luciferase reacts with D-luciferin in the presence of ATP produced by NPP4. The resulting luminescence was measured between 10–14 min at 560 nm using a BMG PheraStar FS plate reader (BMG Labtech GmbH, Ortenberg, Germany).

#### NPP3 and NPP5 Assays

The assays were performed in analogy to published procedures (Blacher et al., 2015). The enzymatic activity of human NPP3 and NPP5 (soluble forms expressed in insect cells and purified as previously described (Lee et al., 2015; Lopez et al., 2020) was measured using 1,*N^6^*-etheno-nicotinamide adenine dinucleotide (ϵ-NAD^+^) as a substrate, which is hydrolyzed to fluorescent 1,*N^6^*-etheno-AMP (ϵ-AMP). The enzymatic reactions were performed in reaction buffer [10 mM *N*-cyclohexyl-2-aminoethanesulfonic acid (CHES), 2 mM CaCl_2_, and 1 mM MgCl_2_, pH 9.0 in H_2_O]. Purified NPP3 (90 ng) or NPP5 (400 ng), 20 µM of *ϵ*-NAD^+^ and 10 *µ*M of the test compound were incubated for 30 min at 37°C. The relative fluorescence at 270 nm excitation and 420 nm emission was detected by a fluorescence microplate reader (Flexstation, Medical Devices LLC. USA, Softmax Pro software to collect the data).

#### CD38 Assay

The assay operation was analogous to the NPP3 and NPP5 assays. The enzymatic reactions were performed in 10 mM HEPES reaction buffer (pH 7.2) using 8 ng of human CD38 (expressed in Sf9 insect cells) in analogy to a published procedure (Blacher et al., 2015).

### Metabolic Stability

The experiments were performed by Pharmacelsus, Saarbrücken, Germany (https://www.pharmacelsus.com/services/in-vitro-adme/) using human and mouse liver microsomes (0.5 mg/mL, mixed gender, pooled). Compounds were tested at a concentration of 1 μM. Data points represent means of two separate experiments performed in duplicates.

### Molecular Modeling and Docking Studies

Recently, we reported a homology model of the human CD39 generated based on rat CD39 (PDB ID: 3ZX3, 1.70 Å) to understand the binding mode of the natural substrate ATP and the fluorescent-labeled ATP derivative, PSB-170621A (Lee et al., 2018). The generated homology model of human CD39 was used for the docking procedure using AutoDock 4.2 (Morris et al., 2009). For docking studies on human CD73 we used the recently published X-ray structure of human CD73 (PDB ID: 6S7F, 2.05 Å) co-crystallized with the inhibitor PSB-12379 (Bhattarai et al., 2019). The AutoDockTools (ADT) from Molecular Graphics Laboratory (MGL) were employed to generate the input files for both CD39 and CD73 and to analyze the docking results obtained from AutoDock 4.2 (Sanner, 1999). Prior to docking, the three-dimensional energy scoring grids for a box of 60 × 60 × 60 points with a spacing of 0.375 Å were computed. The grids were centered based on the substrate binding site of the enzyme. For each ligand, 50 independent docking calculations using the *var*CPSO-ls algorithm from PSO@Autodock implemented in AutoDock4.2 were performed and terminated after 500,000 evaluation steps (Namasivayam and Günther, 2007). The parameters of *var*CPSO-ls algorithm, the cognitive and social coefficients c1 and c2, were set at 6.05 with 60 individual particles as a swarm size. Default values were applied for all the other available parameters for the grid generation and docking calculation. The top-scoring binding poses with the lowest energy and highly populated poses were visually analyzed and selected the final binding pose.

## Results and Discussion

### Chemistry

The ATP analog ARL67156 (I), which is known as a standard inhibitor of CD39, was selected as a lead structure, and different substitutions of the adenine base and modifications of the phosphate chain were performed. The appropriate adenosine derivatives were synthesized and subsequently submitted to phosphorylation according to the Ludwig procedure (Ludwig, 1981) with small modifications.

#### Synthesis of Nucleosides

**Scheme 1:**
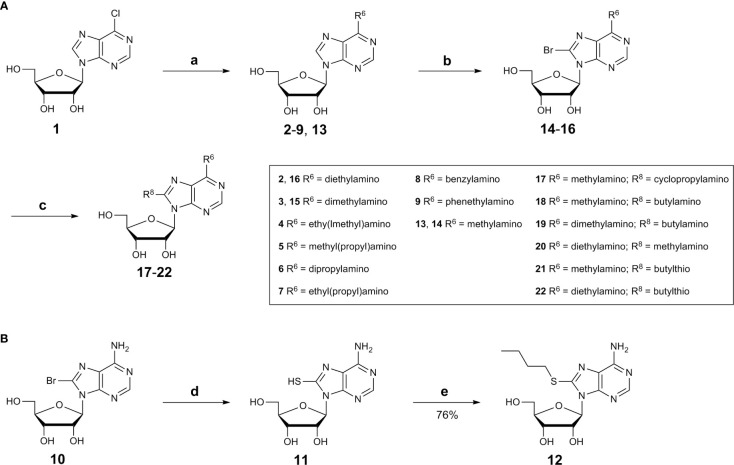
**(A)** Synthesis *N^6^*,8-disubstituted adenosine derivatives (see **Table 1**), Reagents and conditions: a) dialkylamine, Et_3_N, absolute EtOH, reflux, 2-48h; b) bromine, sodium acetate buffer, pH 4.0, room temperature, overnight; c) alkylamine, Et_3_N, absolute EtOH, reflux, 18–48 h; **(B)** Synthesis of 8-substituted adenosine derivatives 11 and 12. Reagents and conditions: d) thiourea, EtOH, 1h, reflux; e) 1-iodobutane, H_2_O/EtOH (1:1), 2 M aq.NaOH.

Adenosine derivatives were synthesized starting with substitutions of the *N^6^*-position. Commercially available 6-chloropurine riboside (1) was reacted with dialkylamine derivatives in the presence of a base in ethanol (**Scheme 1**) (Bhattarai et al., 2015). Purification by silica gel chromatography yielded the desired *N^6^*-disubstituted adenosine derivatives (2–9, 13).

Since 8-BuS-AMP (II), 8-BuS-ADP and 8-Bu-ATP were described as CD39 inhibitors (Lecka et al., 2013), we introduced an 8-butyl substituent to study its effect on the ATP analogs as well. For this purpose, 8-bromoadenosine (10) was reacted with thiourea in ethanol yielding the intermediate 8-thioadenosine (11), which was subsequently alkylated using 1-iodobutane in a mixture of water and ethanol (1:1) in the presence of sodium hydroxide (**Scheme 1**) (Fox et al., 1958; Kikugawa et al., 1973; El-Tayeb et al., 2009). Purification by silica gel chromatography yielded the desired adenosine derivative 12.

In order to investigate whether 8- and *N^6^*-substitution could be additive, combinations of both were synthesized. For this purpose, *N^6^*-substituted adenosine derivatives (2, 3, and 13) were prepared as described above in **Scheme 1** (Bhattarai et al., 2015). Then, the 8-position was brominated under acidic conditions (Ikehara and Uesugi, 1969; Bhattarai et al., 2015). The pH value of the reaction was maintained by adding 0.1 M sodium acetate buffer (pH 4.0). Excess bromine was subsequently removed by sodium hydrogen sulfite, and neutralization with aqueous NaOH solution followed by filtration affording the desired compounds 14–16 (**Scheme 1**). The bromine atom was subsequently substituted by an alkylamine to obtain compounds 17–22 (**Scheme 1**) (Long et al., 1967; Chattopadhyaya and Reese, 1977; Bhattarai et al., 2015).

#### Synthesis of Nucleotides

**Scheme 2:**
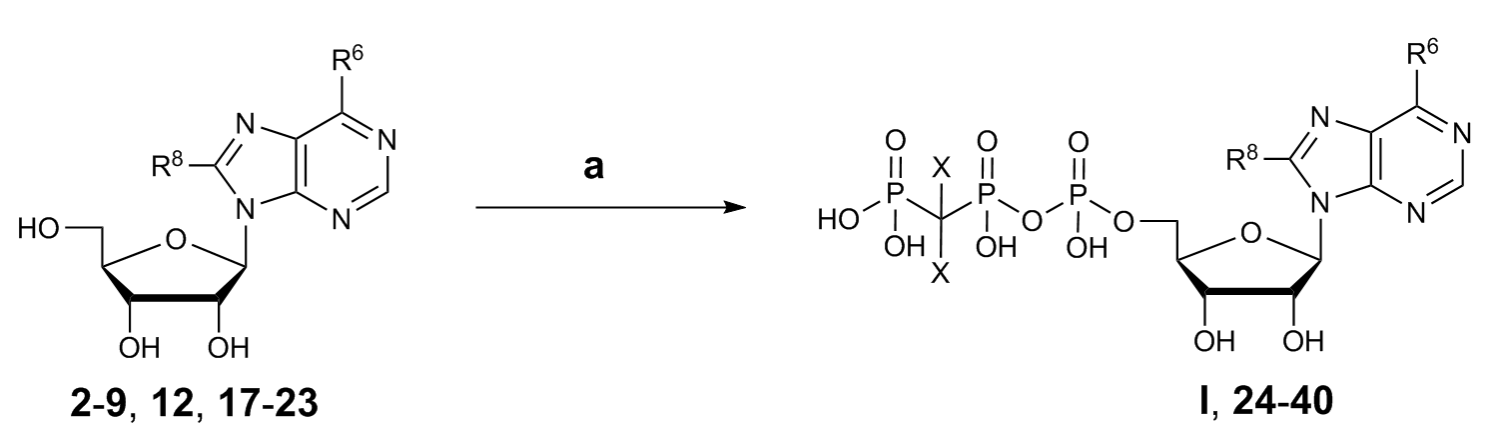
General synthesis of nucleotides I and 24–40 by triphosphorylation. Reagents and conditions: a) three steps: (i) trimethylphosphate, phosphoryl chloride, proton sponge [1,8-bis-(dimethylamino)naphthaline], 0–4°C, 4–5 h, argon; (ii) For 24–38: 0.5 M tris-*N*-butylammonium-dibromomethylene-bisphosphonate [Bu_3_N CBr_2_(PO_3_H)_2_] solution in anhydrous DMF, Bu_3_N, 0–4°C, 5 min. For 39: 0.5 M Bu_3_N·CCl_2_(PO_3_H)_2_ solution in anhydrous DMF, Bu_3_N, 0–4°C, 5 min. For 40: 0.5 M Bu_3_N CF_2_(PO_3_H)_2_ solution in anhydrous DMF, Bu_3_N, 0–4°C, 5 min.; (iii) 0.5 M TEAC buffer pH 7.4–7.6, room temperature, 1 h. For R^6^ and R^8^ see (**Scheme 1**) (2–9, 12, 17–22) and **Table 1** (I, 24–40); compound 23 is adenosine R^6^, R^8^ = H).

The adenosine derivatives were submitted to phosphorylation according to the Ludwig procedure with small modifications (Ludwig, 1981). The lyophilized nucleosides were dissolved in trimethylphosphate and reacted with phosphoryl chloride (POCl_3_) in the presence of proton sponge [1,8-bis-(dimethylamino)naphthaline] to yield the reactive 5’-dichlorophosphates as intermediates (Yoshikawa et al., 1967; El-Tayeb et al., 2009). Reaction with tris-*N*-butylammonium-dibromomethylene-bisphosphonate in anhydrous *N,N-*dimethylformamide (DMF) followed by hydrolysis with triethylammonium hydrogencarbonate (TEAC) buffer led to the desired nucleotide analogs (**Scheme 2**).

Dibromomethylenebisphosphonate was synthesized from tetraisopropyl-methylenebisphosphonate according to published procedures (Mohamady and Jakeman, 2005; McKenna et al., 2007; Oertell et al., 2014). After completion of the phosphorylation reaction, trimethylphosphate was removed from the crude reaction mixture by extraction with *tert*.-butylmethylether followed by lyophilization of the water layer. The nucleotides were purified by anion exchange chromatography on a sepharose column using a fast protein liquid chromatography (FPLC) apparatus by applying a linear gradient (5–80%, 0.5 M aqueous ammonium hydrogencarbonate buffer in water) (McCoy et al., 2014). The neutral impurities (e.g. nucleosides) eluted first, followed by charged species [mono-, di-, and finally triphos(phon)ates]. The products were further purified by HPLC on reverse-phase C18 material to remove inorganic phosphates and buffer components to yield the desired nucleoside triphosphate analogs I and 24–40 in high purity of ≥92% (**Table 1**).

**Table 1:** Potency of nucleotides as inhibitors of human CD39.

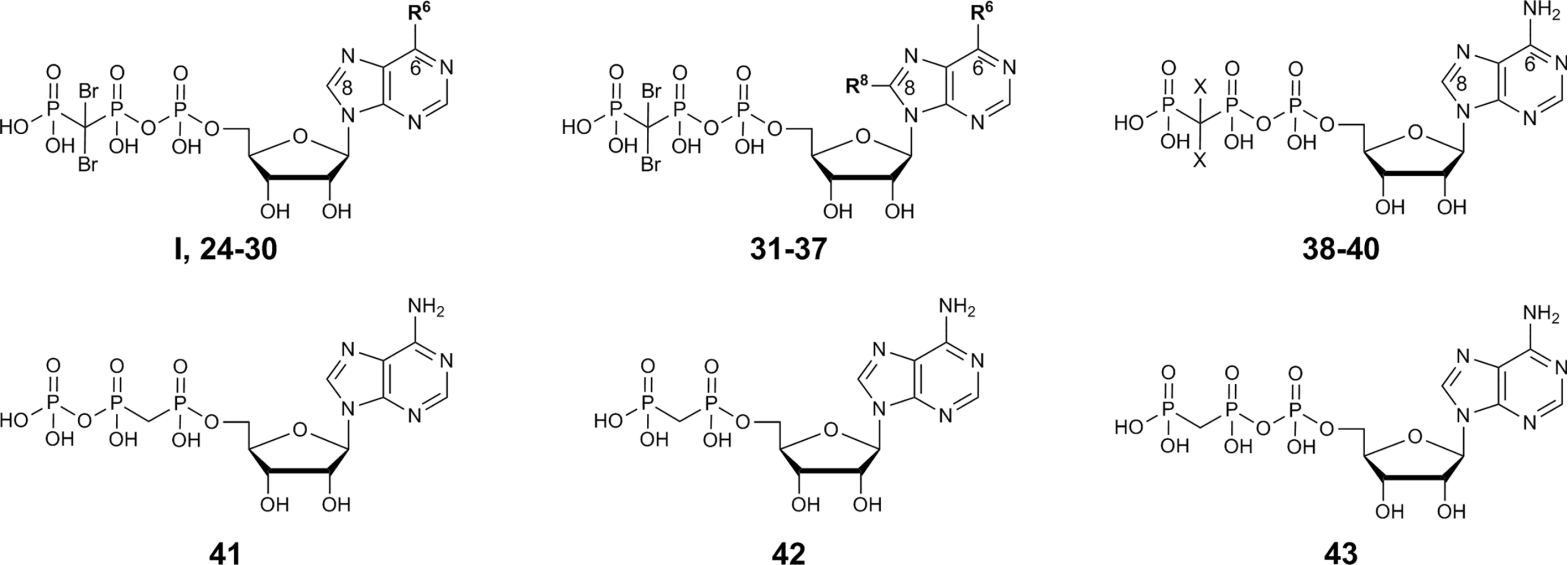			
Compound	Structure		K_i_ ± SEM (µM)^a^^,^^b^ *(or % inhibition at 10 µM)*
**N^6^-Substituted β,γ-dibromomethylene-ATP derivatives I, 24-30**			
	**R^6^**		
**I, ARL67156**	-N(CH_2_CH_3_)_2_		**0.973** ± 0.239 **3.45** ± 0.56^c^
**24**	-N(CH_3_)_2_		**40.1** ± 7.0
**25**	-N(CH_2_CH_3_)CH_3_		**6.48** ± 2.6
**26**	-N((CH_2_)_2_CH_3_)CH_3_		**4.04** ± 2.12
**27**	-N((CH_2_)_2_CH_3_)_2_		**2.68** ± 1.11
**28**	-N((CH_2_)_2_CH_3_)(CH_2_CH_3_)		**2.22** ± 0.02
**29**	-NH-benzyl		>> 10 (4%)
**30**	-NH((CH_2_)_2_Ph)		**4.82** ± 0.21
**N^6^,8-Disubstituted β,γ-dibromomethylene-ATP derivatives 31-37**			
	**R^6^**	**R^8^**	
**31**	-NH_2_	S(CH_2_)_3_CH_3_	**1.13** ± 0.23 **4.11** ± 0.86^c^
**32**	-NHCH_3_	-NH-cyclopropyl	**5.72** ± 0.86
**33**	-NHCH_3_	-NH(CH_2_)_3_CH_3_	**1.51** ± 0.40 **3.35** ± 1.63^c^
**34**	-N(CH_3_)_2_	-NH(CH_2_)_3_CH_3_	≈ 10 (54%)
**35**	-N(CH_2_CH_3_)_2_	-NHCH_3_	≈ 10 (59%)
**36**	-NHCH_3_	-S(CH_2_)_3_CH_3_	> 10 (39%)
**37**	-N(CH_2_CH_3_)_2_	-S(CH_2_)_3_CH_3_	**7.48** ± 1.29 **5.98** ± 5.26^c^
**β,γ-Dihalogenomethylene-ATP analogs 38-40**			
	**X**		
**38**	-Br		**5.26** ± 0.22
**39**	-Cl		**9.53** ± 1.46
**40**	-F		**10.6** ± 0.4 **4.55** ± 0.49^c^
**Methylene-ATP and –ADP analogs 41-43**			
**41** α,β-Methylene-ATP	*see structure above*		**0.632** ± 0.024 **7.20** ± 0.64^c^
**42** α,β-Methylene-ADP (AOPCP)	*see structure above*		>> 10 (14%)
**43** β,γ-Methylene-ATP	*see structure above*		>> 10 (23%)

a Fluorescence capillary electrophoresis assay: screening at 10 µM was performed, or concentration-inhibition curves (n = 3) were determined using the fluorescent substrate PSB-017621A (0.5 µM).

b Ki values are depicted in bold.

c Malachite green assay: concentration-inhibition curves (n = 3) were determined using the natural substrate ATP (50 µM).

For investigating structure-activity relationships regarding the triphosphate moiety, variants of the β,γ-dibromomethylene group are of interest. The naked β,γ-methylene-ATP (43), without any substituents attached to the methylene group, was commercially available. β,γ-Dibromomethylene-ATP (38) was synthesized starting from adenosine (23) according to the procedure described above. Additionally, β,γ-dichloro- and β,γ-difluorobisphosphonic acid were synthesized according to published procedures (McKenna et al., 1988; Boyle, 2006). The bisphosphonic acids were converted to the corresponding tri-*N*-butylammonium salts by dissolution of the acids in 50% aqueous ethanol and subsequent drop-wise addition of tri-*N*-butylamine until a pH of 7.8–8.0 was reached followed by evaporation and lyophilization (McKenna et al., 1988; Oertell et al., 2014). Triphosphorylation reaction with adenosine (23) and subsequent purification was carried out as described above to yield the desired ATP analogs 39 and 40 (**Table 1**). For reference purposes, the lead structure ARL67156 (I) was also synthesized. The structures of the obtained synthesized nucleotide analogs were confirmed by ^1^H-, ^13^C-, and ^31^P-NMR spectroscopy, in addition to LC/ESI-MS analysis performed in both positive and negative mode. Purity was determined by high-performance liquid chromatography (HPLC)-UV (254 nm)-electrospray ionization mass spectrometry (ESI)-MS. NMR and LCMS data of selected final products are depicted in **Figures S1–S5** (see Supplementary Material).

### Biological Evaluation

#### CD39 Inhibition

Inhibition of human CD39 was determined using the previously developed fluorescence-based capillary electrophoresis method utilizing a fluorescent ATP derivative as a substrate (Lee et al., 2018). For compounds showing high inhibition (>60% at 10 µM concentration) concentration-inhibition curves were determined using the same assay. Selected compounds were additionally investigated using the malachite green assay in order to confirm the results using the natural substrate ATP (**Table 1**). ARL67156 had been shown to be a competitive inhibitor (Lévesque et al., 2007), and the same inhibition type can be assumed for its derivatives and analogs, which bear structural resemblance to the CD39 substrate ATP. *K_i_* values were calculated using the Cheng-Prusoff equation (Cheng and Prusoff, 1973).

The lead structure ARL67156 displayed a *K_i_* value of 0.973 µM in our fluorescence-based CE assay, being somewhat more potent than previously reported (Lévesque et al., 2007). In the malachite green assay versus ATP as a substrate, it showed a *K_i_* value of 3.45 µM, which is in the same range. Replacement of one ethyl group by a methyl group at the *N^6^*-nitrogen atom of ARL67156 (I) reduced potency by about 7-fold (compound 25, *K_i_* 6.48 µM), while replacement of both *N^6^*-ethyl groups by methyl in 24 had an even more dramatic effect (*K_i_* 40.1 µM), 41-fold decrease compared to I. Introduction of propyl substitution was better tolerated, see 27 (*N^6^*-dipropyl) and 28 (*N^6^*-ethyl,*N^6^*-propyl-substituted) with *K_i_* values of 2.68 and 2.22 µM, respectively. The *N^6^*-methyl,*N^6^*-propyl derivative 26 was also in the same range as the *N^6^*-methyl,*N^6^*-ethyl derivative 25, indicating that the enzyme accommodates lipophilic substituents in that position. While an *N^6^*-benzyl residue (in 29) led to abolishment of the CD39-inhibiting activity, *N^6^*-phenylethyl-substitution (derivative 30) restored inhibitory activity (*K_i_* 4.82 µM). This might be explained by the higher flexibility of the phenethyl group and its increased lipophilicity, while the benzyl group may produce clashes with the hydrophobic amino acid residues in the binding pocket.

As a next step we investigated 8-substituted analogs of ARL67156 with optional *N^6^*-mono- or disubstitution (compounds 31–37). These compounds were inspired by 8-butylthio-AMP (II) which had been reported as a similarly potent CD39 inhibitor as ARL67156 (Lecka et al., 2013). These nucleotides can be regarded as hybrid molecules derived from I and II, containing features of both CD39 inhibitors. In fact, 2-butylthio-substitution of *N^6^*-unsubstituted I was equally potent as ARL67156 (I) as confirmed in both assays, against fluorescent (*K_i_* 1.13 µM) and natural substrate (*K_i_* 4.11 µM) (compound 31, **Table 1**). However, combination with the *N^6^*-diethyl substitution of I led to significantly reduced potency (37, *K_i_* 7.48 and 5.98 µM in the two employed assays), while the 8-butylthio-*N^6^*-monomethyl-substituted derivative 36 was even less potent (*K_i_* > 10 µM). This indicates that both substituents, at C8 and *N^6^*, have interdependent effects on potency and are not simply additive.

We subsequently replaced the 8-butylthio residue by other 8-substituents connected *via* an amino rather than a thio linker (32–35). The smaller methylamino residue in the 8-position in combination with the *N^6^*-diethyl substitution of I led to reduced potency (compound 35, *K_i_* ≈ 10 µM). However, 8-butylamino substitution in combination with a small *N^6^*-monomethyl residue in 33 again led to a similarly potent CD39 inhibitor as lead structure I and *N^6^*-unsubstituted 8-butylthio derivative 31 (see compound 33, *K_i_* 1.51 and 3.35 µM in the two employed assays). A cyclopropylamino residue in the 8-position was not superior but resulted in a slight reduction in potency (compare 32 and 33). Introduction of a second *N^6^*-methyl group into 33 reduced the potency (34, *K_i_* ≈ 10 µM).

**Figure 3:**
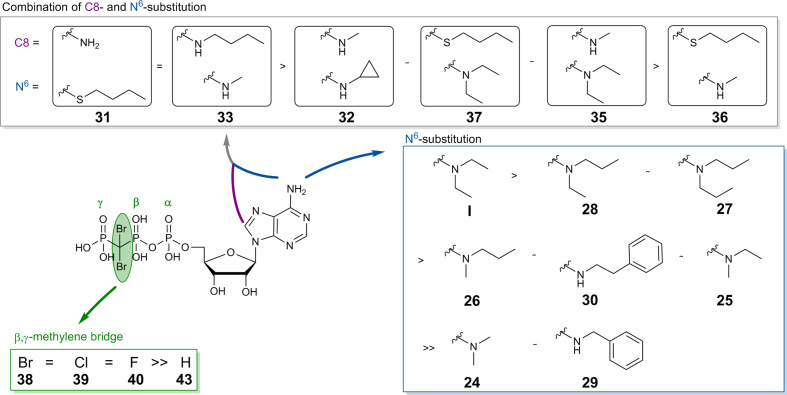
Structure-activity relationships of ARL67156 (**I**) derivatives and analogs as CD39 inhibitors.

With a further set of compounds, we investigated the replacement of the dibromo-substitution on the triphosphate-analogous linker of lead structure I. For simplification, we prepared the corresponding *N^6^*-unsubstituted analogs. The direct *N^6^*-unsubstituted analog of I, compound 38, with dibromomethylene modification of the triphosphate chain, was about 5-fold less potent than the lead compound I (*K_i_* 5.26 µM vs. 0.973 µM). Its dichloro- (39) and difluoro-substituted (40) analogs were only about 2-fold less potent than the more lipophilic dibromo-derivative 38, while the unsubstituted β,γ-methylene-ATP (43) was virtually inactive. These results indicate that an electron-withdrawing substituent on the β,γ-methylene-ATP derivatives was required. In the CD39 substrate ATP and in the inhibitor α,β-methylene-ATP (41), which is a poor substrate of CD39, the β,γ-oxygen bridge exerts electron withdrawing effects. In fact, 41 was found to be as potent as ARL67156 (I) in blocking CD39 (*K_i_* 0.632 µM vs. the fluorescent substrate). It was less potent in the malachite green assay, perhaps due to partial hydrolysis during the longer incubation time in that assay (3 vs. 15 min). The structure-activity relationships of all ARL67156 (I) derivatives and analogs are represented in **Figure 3**.

#### Selectivity

**Figure 4:**
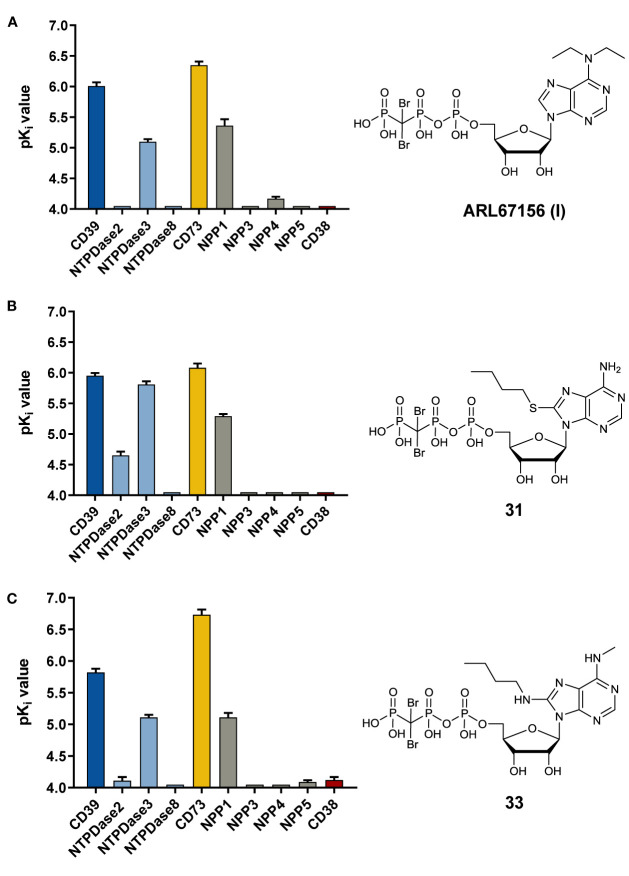
Selectivity profile of selected CD39 inhibitors **(A)** Effect of ARL67156 (I), **(B)** compound 31 and **(C)** compound 33 at human ecto-nucleotidases. p*K_i_* values for CD39, as determined in the fluorescence capillary electrophoresis assay, are compared to those at other ecto-nucleotidases. Assay procedures are described in the method section.

ARL67156 was previously described as a competitive inhibitor of CD39 (*K_i_* = 11 ± 3 µM), NTPDase3 (*K_i_* = 18 ± 4 µM), and NPP1 (*K_i_* = 12 ± 3 µM) (Lévesque et al., 2007). In the present study, the selectivity of ARL67156 (I) and its analogs was assessed by testing lead structure I and the two most potent derivatives 31 and 33 in a large array of human ectonucleotidases, namely NTPDases1 (CD39), -2, -3, and -8, NPP1, -3, -4, and -5, CD73 (ecto-5’-nucleotidase) and CD38 (for results see **Table 2** and **Figure 4**). The experiments were performed by established procedures (Freundlieb et al., 2014; Lee et al., 2015; Blacher et al., 2015; Lopez et al., 2020). All of the compounds inhibited also NTPDase3, CD73 and NPP1, but they showed lower potency at NTPDase3 and NPP1 than at CD39. The inhibition of CD73 was equal to that of CD39, with the exception of compound 33, which inhibited CD73 with an even 8-fold higher potency compared to CD39. Compound 31 was found to also weakly inhibit NTPDase2.

**Table 2:** Inhibition of selected ecto-nucleotidases by ARL67156 (I) and analogs 31 and 33^a^.

Enzyme	*K_i_* ± SEM (µM) *(or % inhibition)*		
	ARL67156 (I)	31	33
**CD39**	**0.973** ± 0.239	**1.13** ± 0.23	**1.51** ± 0.40
**NTPDase2**	> 50 (18%)	**22.2** ± 0.5	**78.0** ± 0.6
**NTPDase3**	**7.94** ± 1.36	**1.54** ± 0.34	**7.80** ± 1.34
**NTPDase8**	> 50 (2%)	> 50 (2%)	> 50 (-11%)
**CD73**	**0.451** ± 0.121	**0.831** ± 0.274	**0.185** ± 0.074
**NPP1**	**4.41** ± 3.53	**5.17** ± 1.75	**7.68** ± 5.40
**NPP3**	> 10 (13%)	> 10 (8%)	> 10 (8%)
**NPP4**	> 10 (-1%)	> 10 (2%)	> 10 (4%)
**NPP5**	> 10 (-3%)	> 10 (2%)	> 10 (1%)
**CD38**	> 10 (0%)	> 10 (7%)	> 10 (6%)

a K_i_ values of potent compounds are shown in bold.

The selectivity data clearly shows that the reported CD39 inhibitor ARL67156 (I), which is commercially available and broadly used in biological studies, is in fact a dual CD39/CD73 inhibitor showing ancillary inhibition of NPP1 and NTPDase3 at higher concentrations. 8-Butylthio-β,γ-bromomethylene-ATP (31) displays a similar profile with comparable potency at CD39. Both compounds could, in fact, be characterized as multi-target ectonucleotidase inhibitors. Compound 33 with an *N^6^*-methyl residue and 8-butylamino-substitution is even significantly more potent as inhibitor of ecto-5’-nucleotidase (CD73, *K_i_* 0.185 µM, 8-fold difference) than of CD39. All three inhibitors could serve as novel lead structures for developing dual CD39/CD73 inhibitors or triple CD39/CD73/NPP1 inhibitors which might be advantageous for the immunotherapy of cancer as compared to selective inhibitors that block only a single ectonucleotidase.

#### Metabolic Stability

The most potent CD39 inhibitors I, 31, and 33 were further studied for metabolic stability in human and mouse liver microsomes which are mainly responsible for drug metabolism (see **Figure S8**). Surprisingly, all three compounds appeared to be metabolically highly unstable with half-lives of less than 1 min. To ensure that degradation was caused by microsomal enzymes and not due to chemical instability, stock solutions were analyzed by LC/ESI-MS analysis, and were in all cases found to be stable. ARL67156 (I) is commonly used as a “selective” CD39 inhibitor, and the compound had been assumed to be metabolically stable in biological studies because of its β,γ-dibromomethylene bridge (Crack et al., 1995; Lévesque et al., 2007). However, the present results show that ARL67156 and its derivatives are not suitable for *in vivo* application. Nevertheless, they represent useful tool compounds for *in vitro* studies.

#### Molecular Modelling Studies

##### NTPDase1 (CD39)

Recently, we published a homology model of human CD39 based on the crystal structures of rat CD39 and human NTPDase2 (Lee et al., 2018). In the present study, we utilized this model for docking studies to rationalize the observed SARs. As a competitive inhibitor, ARL67156 (I) binds to the catalytic site of the enzyme and is predicted to possess virtually the same orientation and similar interactions as the natural substrate ATP. The key interactions of ATP with the amino acid residues in the binding site of CD39 had previously been verified by mutagenesis studies as discussed by Lee et al. (2018).

**Figure 5:**
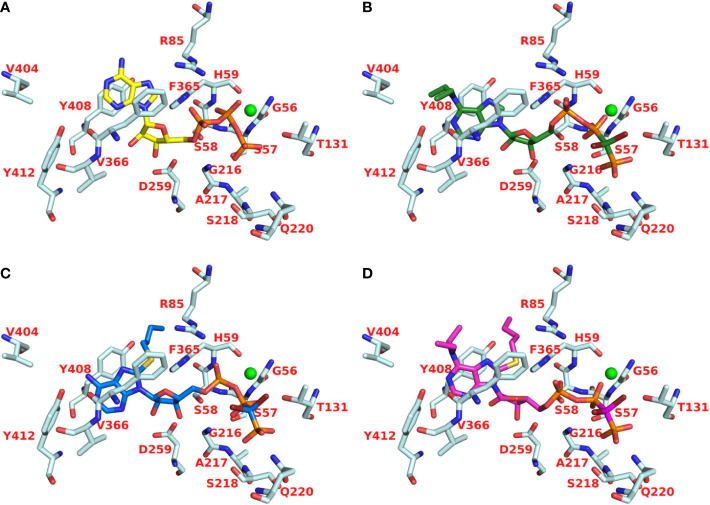
Docked poses nucleotides in the substrate binding pocket of the human CD39 homology model. Binding poses of the natural substrate ATP (yellow) **(A)**, of **I** (green) **(B)**, of **31** (marine blue) **(C)**, and of **37** (magenta) **(D)** are shown; the important amino acids are colored in pale cyan and shown in stick representation. The cofactor Ca^2+^ is represented as a green sphere. Oxygen atoms are colored in red and nitrogen atoms in blue.

In brief, the α-phosphate group of ATP (**Figure 5A** and **Figure S9A**) interacts with H59, the β-phosphate group with G56, S57, and S58, while T131, G216, A217, and S218 form interactions with the γ-phosphate, either directly, or mediated by water. The calcium cation forms an octahedral complex and stabilizes the phosphate groups in the binding pocket *via* interactions with the β- and γ-phosphates. The 3’-hydroxy-group of the ribose interacts with D259, while the adenine ring is sandwiched between F365 and Y408 and stabilized by π-π-interactions.

ARL67156 (I, **Figure 5B**) was docked and found to have a similar orientation in the binding site of the enzyme as ATP. The key residue interactions of the phosphate groups, the hydroxy groups of the ribose and the adenine ring are identical for ATP and ARL67156. The dibromomethylene substitution prevents hydrolysis by the enzyme and additionally ensures full deprotonation of the γ-phosphate due to its electron-withdrawing properties. Unsubstituted β,γ-methylene-ATP (43) shows no significant inhibition, while the halogen-substituted ATP analogs 38, 39, and 40 inhibit the enzyme with IC_50_ values in the low micromolar range. Full deprotonation of the γ-phosphate likely favors interactions with the amino acid residues, the main chain of G216, A217, S218 and the side chain of Q220 in the binding pocket, and their interaction with the calcium ion (see **Figure S9**).

The putative binding pose of the natural substrate ATP (**Figure 5A**) shows that the amino group in the C6-position does not appear to directly interact with amino acid residues of the enzyme; it is oriented towards the surface of the enzyme. This surface of the binding pocket is lined by a large number of hydrophobic residues, F365, V366, V404, Y408, and Y412. The docked pose of I (ARL67156, **Figure 5B**) suggests that the diethyl substitution at *N^6^* possibly forms hydrophobic interactions with these residues and stabilizes the adenine ring and the phosph(on)ate groups in the binding pocket. This was supported by comparing the biological activity of I (K_i_ = 0.973 µM) with the analogous compound with an unsubstituted amino group (38, *K_i_* = 5.26 µM). However, substitution with shorter (24) or larger (25–28) alkyl chains resulted in a decrease in inhibitory potency in comparison to I. A phenethyl residue was tolerated (compound 30, *K_i_* = 4.82 µM) while a benzyl group (29) was not. This may be due to the lower flexibility of the benzyl compared to the phenethyl moiety, which may result in clashes with hydrophobic residues such as V404 and others in the binding sub-pocket. This shows that the surface of the binding pocket requires an optimal substitution, a diethyl group as in I, to form hydrophobic interactions.

The putative binding poses of 31 and 37 (**Figures 5C, D** **)** observed in the docking studies show that the butylthio-substitution at position 8 is oriented towards the amino acid residues H59, R85, F365, and Y408. The combination of butylthio at position 8 with an unsubstituted amino group at position 6 (31, K_i_ = 1.13 µM) gave a similarly potent inhibitor as lead structure I (ARL67156, K_i_ = 0.973 µM). The docked pose of 31 (**Figure 5C**) shows the butylthio to be flexible and positioned within the limited available space in the binding pocket. The potency was maintained with a methylamino-substitution at C6 and butylthio replaced with a butylamino residue at the 8-position. However, the potency was decreased upon *N^6^*-diethylamino substitution in 37. Although the docked pose of 37 displayed only minor differences in the orientation in comparison to those of I and 31 in the binding pocket, the two larger substituents might introduce significant differences in the compounds’ conformations and their interaction with amino acid residues in the binding pocket (**Figure 5D**). These results were supported by compounds 34 and 35 in which both positions, *N^6^* and C8, were substituted with larger alkyl residues leading to significantly reduced inhibitory potency at CD39. 2D-interaction diagrams are depicted in **Figure S9** of Supplementary Material.

##### Ecto-5’-Nucleotidase (CD73)

**Figure 6:**
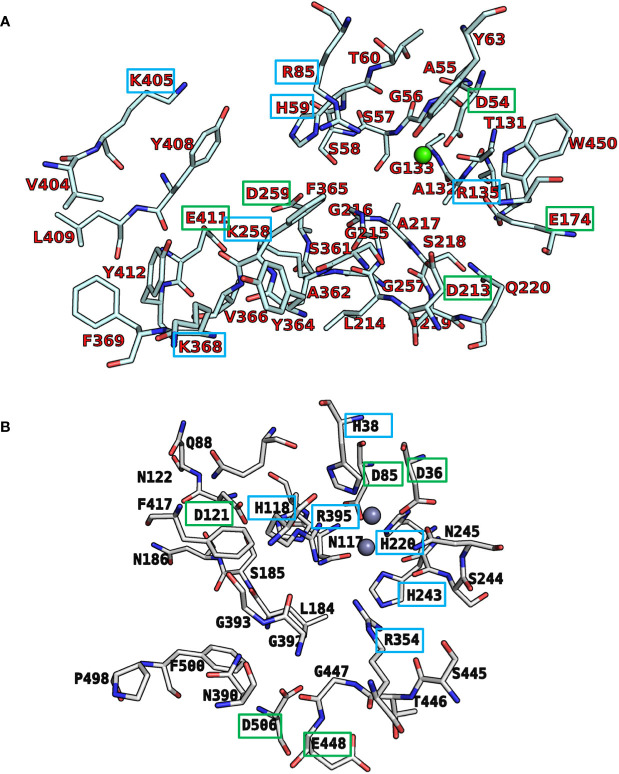
Comparison of **(A)** the putative substrate binding site of human CD39 (pale cyan) and **(B)** the substrate binding site of human CD73 (gray). Important amino acids are shown; positively and negatively charged amino acids are highlighted by blue and green boxes, respectively. Oxygen atoms are colored in red, nitrogen atoms in blue, sulfur atoms in yellow, calcium atom in green, and zinc atoms in dark gray.

**Figure 7:**
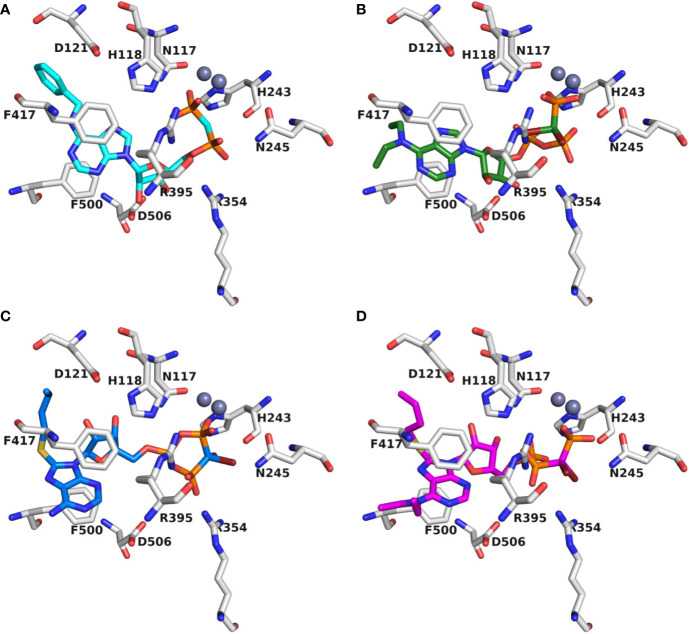
Docked poses of ARL67156 (I) and derivatives in the substrate binding pocket of human CD73 based on an X-ray structure (PDB ID: 6s7f). **(A)** Binding pose of the co-crystallized inhibitor PSB-12379 (orange); **(B)** binding pose of I (green); **(C)** binding pose of 31 (marine blue), and **(D)** binding pose of 37 (magenta). Important amino acids are colored in orange and shown in line representation. The two zinc ions in the substrate binding site are represented as blue spheres. Oxygen atoms are colored in red and nitrogen atoms in blue.

Recently, a high resolution X-ray structure of human CD73 in complex with a subnanomolar inhibitor, the nucleotide analog PSB-12379, derived from the ADP analog AOPCP (or α,β-methylene-ADP) was obtained (Bhattarai et al., 2019). Compared to the human CD39 sequence which consists of 428 amino acids, human CD73 is larger consisting of 574 amino acid residues. The number of positively and negatively charged amino acid residues in the binding pocket are similar in both CD39 and CD73 with six and five, respectively (**Figure 6**). This suggests that the potency of the nucleotide analogs depends on their orientation and interaction with these amino acid residues in the binding pocket. At human CD73, *N^6^*-substituted adenine nucleotide analogs show higher inhibitory potency compared to their *N^6^*-unsubstituted derivatives. PSB-12379 occupies the binding site of the CD73 substrate. The diphosphonate chain (PCP) is bound between the two zinc ions and form electrostatic interactions, the α-phosphonate forms hydrogen bond interactions with N245, R354, and R395, the β-phosphonate group with N117, H118, and R395, and the ribose hydroxyl groups with R354, R395, and D506. The adenine ring is stacked between F417 and F500 (**Figure 7A** and **Figure S10**).

In our selectivity studies, ARL67156 (I) and its derivatives 31 and 33 were found to be similarly or even more potent inhibitors of CD73 as compared to CD39. In order to gain insights into the binding mode of the selected compounds I, 31, and 37, we docked these three inhibitors into the binding site of human CD73. The docked poses of ARL67156 and its derivatives (31 and 37) indicate that the phosphate groups are directing the compounds into the binding pocket and are likely bound between the two zinc ions. Compared to the diphosphonate PSB-12379, the β,γ-methylene triphosphate chain of ARL67156 and its derivatives was observed to be folded inside the binding pocket. In the pocket of CD73, the γ-phosphonate group likely interacts with N117, H118, and R395, the β-phosphonate group with R354 and the α-phosphate with H243 (**Figure 7B**). The hydroxyl groups of the ribose moiety interact with D506 and the adenine ring is likely sandwiched between F417 and F500. The diethylamino substitution at position 6 is extended towards the surface. Interestingly, the butylthio-substituent at position 8 of the adenine ring in compound 31 induces a conformational rotation along the nucleosidic bond shifting the adenine riboside conformation from anti to syn (**Figure 7C**). The hydroxyl groups are predicted to form an interaction with D121. The butylthio group is oriented towards the surface of the enzyme. As shown in **Figure 7D**, the rotation of the ribose is altered and shifted when a large lipophilic alkyl group is introduced as in 37 together with the butylthio group at position 8. 2D-interaction diagrams are depicted in **Figure S10** of Supplementary Material.

## Conclusions

ARL67156 is so far the only commercially available “selective” inhibitor of CD39. Apart from reports describing it as a competitive inhibitor, its characterization has been limited. In the present study we synthesized ARL67156 analogs and derivatives to get insights into the structure-activity relationships of this class of CD39 inhibitors. The presence of electron-withdrawing groups adjacent to the terminal phos-ph(on)ate was found to be crucial indicating that full deprotonation is required for interactions within the orthosteric binding site. The size and polarity of substituents on the adenine ring are required to position it within the apolar substrate binding site of the enzyme. ARL67156 and two of the most potent analogs, 31 and 33, were extensively characterized. Surprisingly, all three CD39 inhibitors were found to be similarly potent or even more potent in inhibiting CD73 and can therefore be envisaged as dual- or multi-target drugs. Dual inhibition of these enzymes, both of which have been proposed as novel targets for cancer immunotherapy, might result in synergistic effects. Both enzymes are cooperating leading to the conversion of proinflammatory ATP to antiinflammatory adenosine. If both, CD39 and CD73, are inhibited at the same time, the concentration of ATP will be increased (by CD39 inhibition), while the concentration of adenosine will be decreased (by CD73 inhibition). This is expected to result in a dramatic enhancement of immunostimulatory, anti-metastatic, and cytotoxic effects (Allard et al., 2017).

However, metabolic stability investigated in human and mouse liver microsomal preparations, was found to be extremely poor, prohibiting their use for *in vivo* studies. Nevertheless, these ectonucleotidase inhibitors should be useful as pharmacological tool compounds for simultaneous inhibition of the CD39/CD73 catalysis cascade *in vitro*. The presented results provide a solid basis for future optimization of nucleotide analogs as CD39 and dual CD39/CD73 inhibitors.

## Data Availability Statement

The raw data supporting the conclusions of this article will be made available by the authors, without undue reservation, to any qualified researcher.

## Author Contributions

LS and CM wrote the manuscript with contributions from all coauthors. CS synthesized most of the compounds. TV synthesized some of the compounds. LS, RI, XL, S-YL, VL, and SM tested the compounds at ectonucleotidases. JP and JS produced the preparations of CD39 and other recombinant NTPDases. VN and LS performed the molecular modeling studies. CM designed and supervised the project.

## Funding

Funded by the Deutsche Forschungsgemeinschaft (DFG, German Research Foundation) - Project-ID: 335447717 - SFB 1328. JS received support from the Natural Sciences and Engineering Research Council of Canada (NSERC; RGPIN-2016-05867) and was the recipient of a “Chercheur National” Scholarship from the Fonds de Recherche du Québec – Santé (FRQS).

## Conflict of Interest

The authors declare that the research was conducted in the absence of any commercial or financial relationships that could be construed as a potential conflict of interest.

## Abbreviations

ADT, AutoDockTools; CE, capillary electrophoresis; DCM, dichloromethane; DMSO, dimethyl sulfoxide; DAD, diode array detector; CD39, nucleoside triphosphate diphosphohydrolase1; CD73, ecto-5’-nucleotidase; ESI, electrospray ionization; FPLC, fast protein liquid chromatography; HEPES, 4-(2-hydroxyethyl)piperazine-1-ethanesulfonic acid; HPLC, high performance liquid chromatography; LIF, laser-induced fluorescence; MS, mass spectrometry; MGL, Molecular Graphics Laboratory; DMF, *N*,*N*-dimethylformamide; NMR, nuclear magnetic resonance; NTPDase1, nucleoside triphosphate diphosphohydrolase1; NPP1, nucleotide pyrophosphatase/phosphodiesterase1; SAR(s), structure-activity relationship(s); TEAC, triethylammonium hydrogencarbonate; TLC, thin layer chromatography.
