# Supplementary material for: Nucleotide Analog ARL67156 as a Lead Structure for the Development of CD39 and Dual CD39/CD73 Ectonucleotidase Inhibitors
Source: Front Pharmacol. 2020 Sep 8;11:1294. doi: 10.3389/fphar.2020.01294 (PMC7508162; doi:10.3389/fphar.2020.01294)
Supplement: Supplementary file 1 [file DataSheet_1.pdf]

Supplementary Material

Nucleotide Analog ARL67156 as a Lead Structure for the Development of CD39 and Dual CD39/CD73 Ectonucleotidase Inhibitors

Laura Schäkel[1,#], Constanze C. Schmies[1,#], Riham M. Idris[1], Xihuan Luo[1], Sang-Yong Lee[1], Vittoria Lopez[1], Salahuddin Mirza [1], The Hung Vu[1], Julie Pelletier[2], Jean Sévigny[2,3], Vigneshwaran Namasivayam[1] and Christa E. Müller [1,*]

[1] PharmaCenter Bonn, Pharmaceutical Institute, Pharmaceutical Sciences Bonn (PSB), Pharmaceutical & Medicinal Chemistry, University of Bonn, Germany

[2] Centre de Recherche du CHU de Québec – Université Laval, Québec City, QC, Canada

[3] Départment de Microbiologie-Infectiologie et d'Immunologie, Faculté de Médecine, Université Laval, Quebec City, QC, Canada

[#] These authors contributed equally to this work

*** Correspondence:**

Christa E. Müller
Pharmaceutical Institute, Pharmaceutical & Medicinal Chemistry,
An der Immenburg 4, D-53121 Bonn, Germany
E-mail: christa.mueller@uni-bonn.de
Tel: +49-228-73-2301
Fax: +49-228-73-2567

Table of contents

**Contents**	**Page**
**Figures S1-S2**. NMR data of selected compounds	S2-S3
**Figures S3-S5**. LC-MS data of selected compounds	S4-S6
**Figures S6-S7** MS data with isotope distribution of selected compounds	S7-S8
**Figure S8.** Metabolic stability of selected compounds	S9
**Figure S9**. 2D-interaction diagrams of nucleotides in the binding site of human CD39	S10-S11
**Figure S10**. 2D-interaction diagrams of nucleotides in the binding site of human CD73	S12-S13

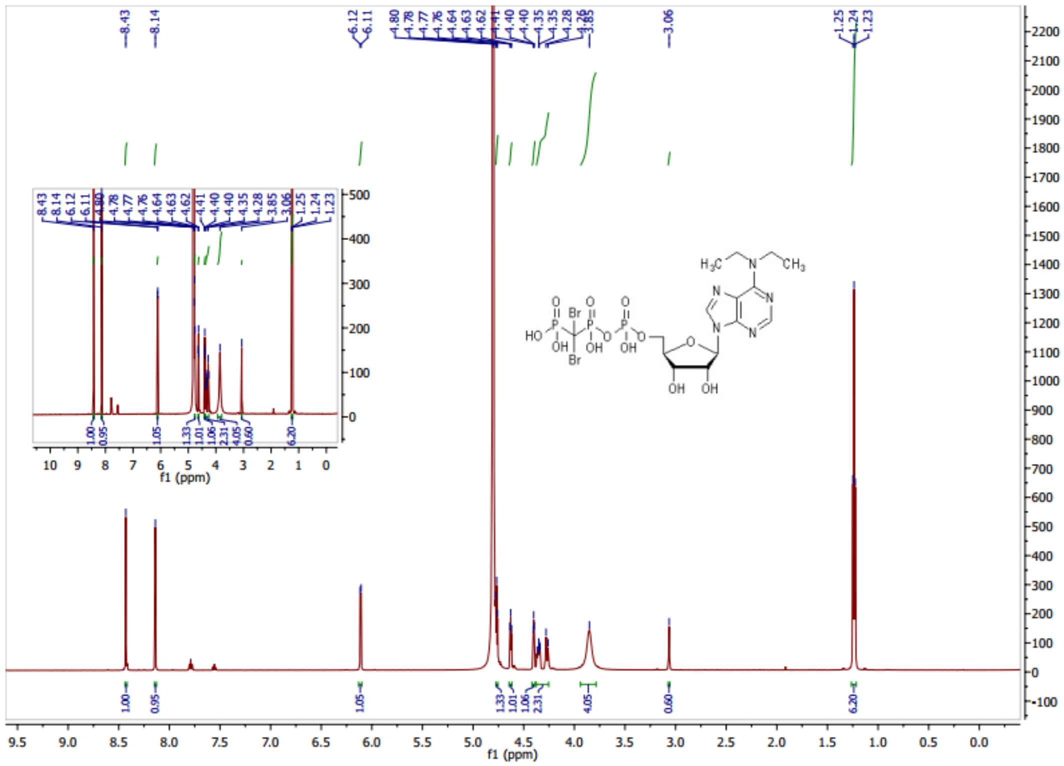

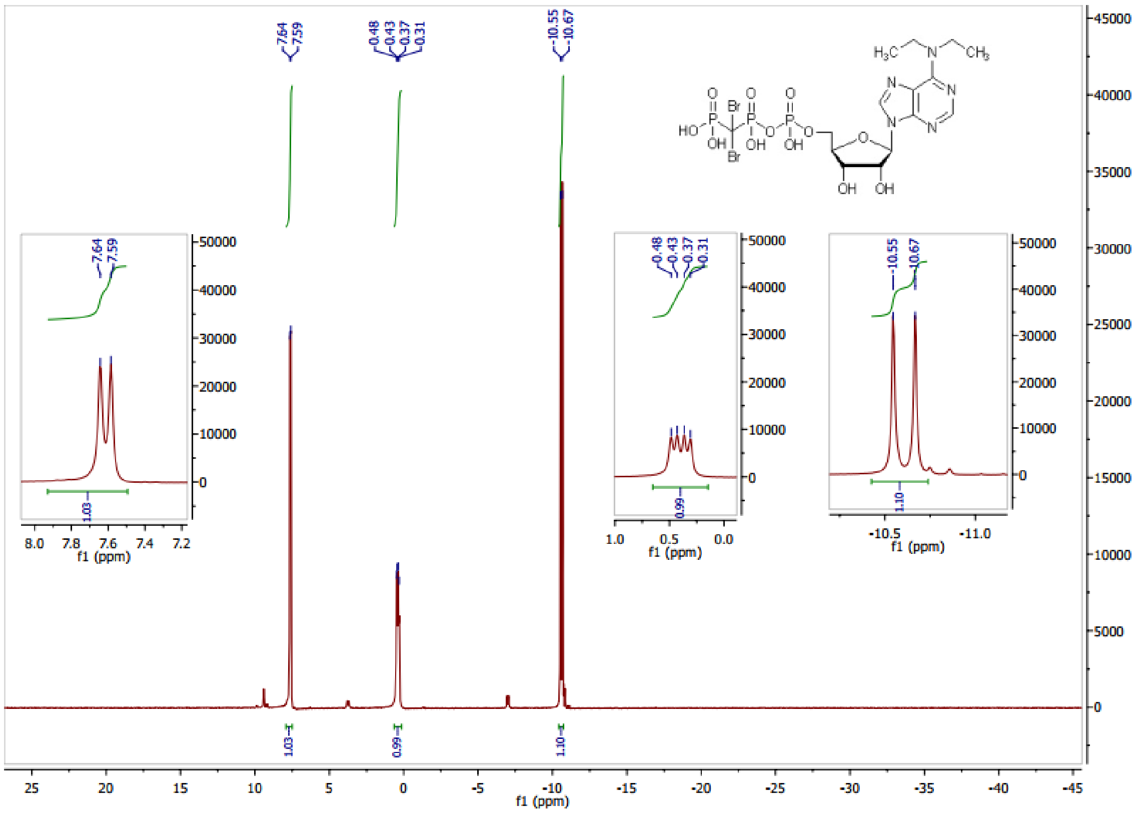

Figure S1. ^{1}H (500 MHz) and ^{31}P (202 MHz) spectra of synthesized ARL67156 (**I**)

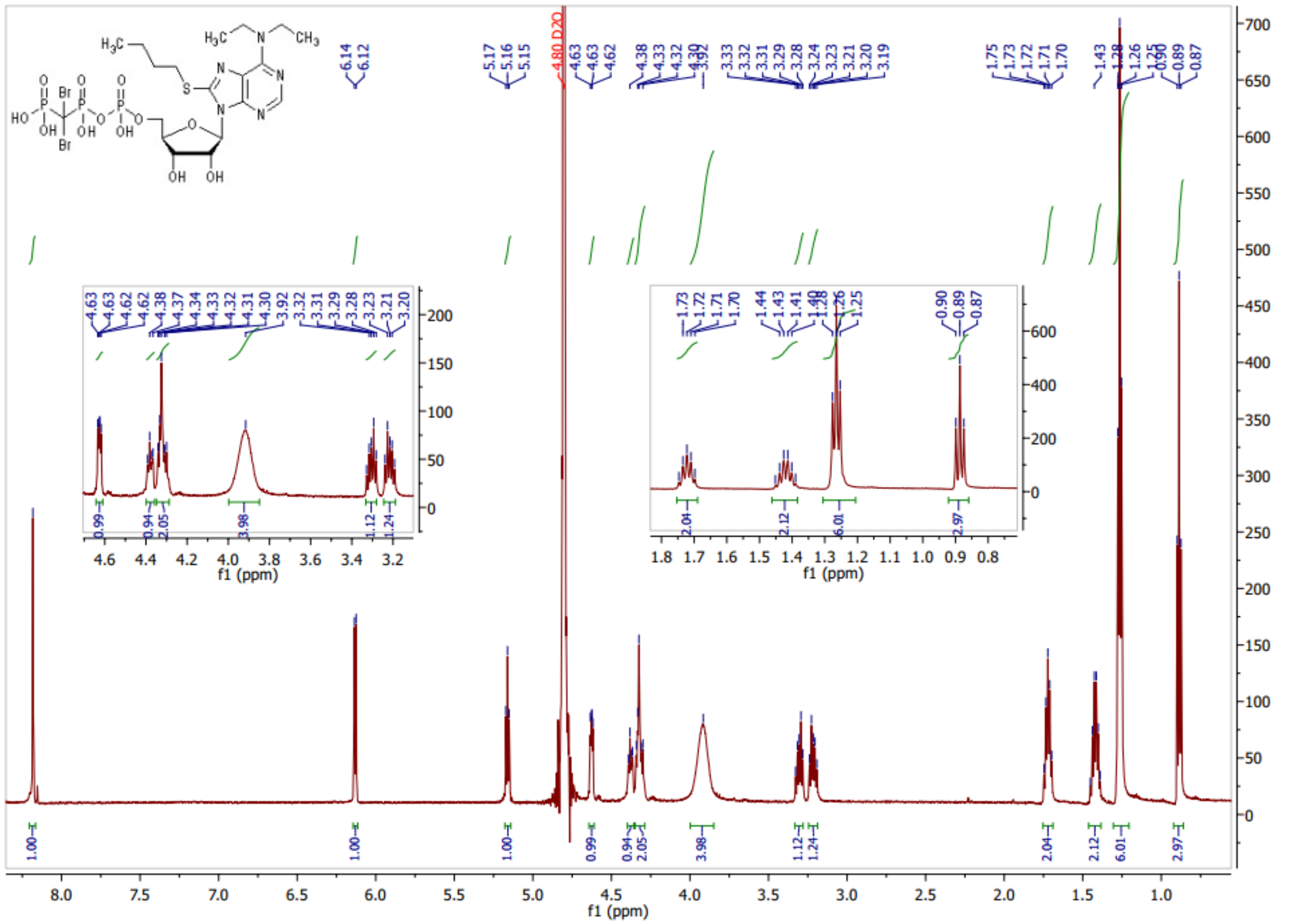

Figure S2. ^{1}H NMR spectrum (500 MHz) of (dibromo((((((2*R*,3*S*,4*R*,5*R*)-5-(8-(butylthio)-6-(diethylamino)-9*H*-purin-9-yl)-3,4-dihydroxy-tetrahydrofuran-2-yl)methoxy)(hydroxy)phosphoryl)-oxy)(hydroxy)phosphoryl)methyl)phosphonic acid (**37**)

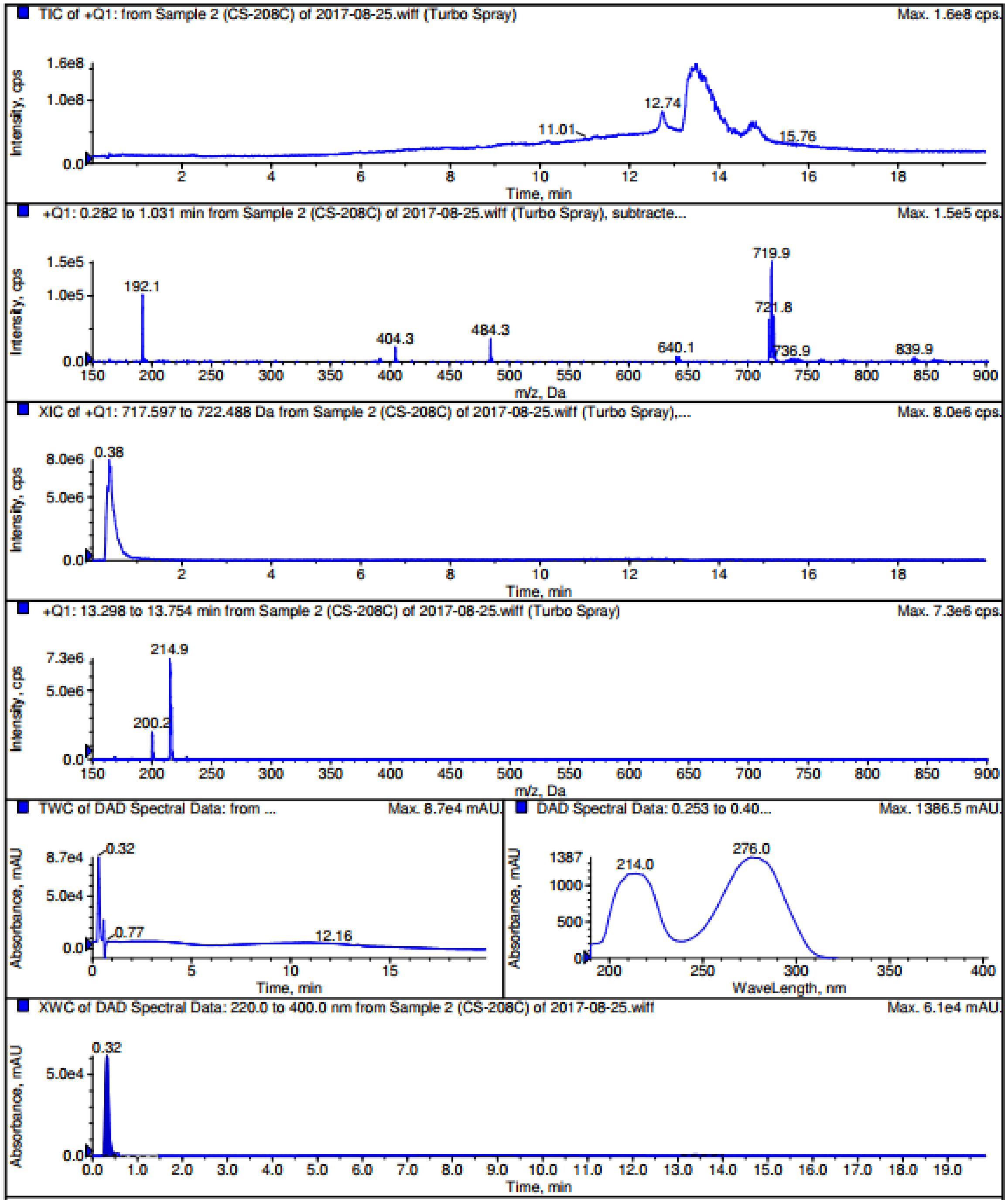

Peak List for "XWC of DAD Spectral Data: 220.0 to 400.0 nm from Sample 2 (CS-208C) of 2017-08-25.wiff"

	Time (min)	Area (mAU x min)	% Area	Height (mAU)	% Height	Width (min)	Baseline Type
1	0.3229	3.3076e5	92.5423	6.1080e4	97.9697	0.3000	Base to Base
2	0.7026	1.7878e4	5.0021	756.5644	1.2135	0.8200	Base to Base
3	13.3892	8776.8639	2.4556	509.2772	0.8169	0.9267	Base to Base

Figure S3. LC-MS spectrum of synthesized ARL67156 (**I**). The purity of compound **I** was 97.5%

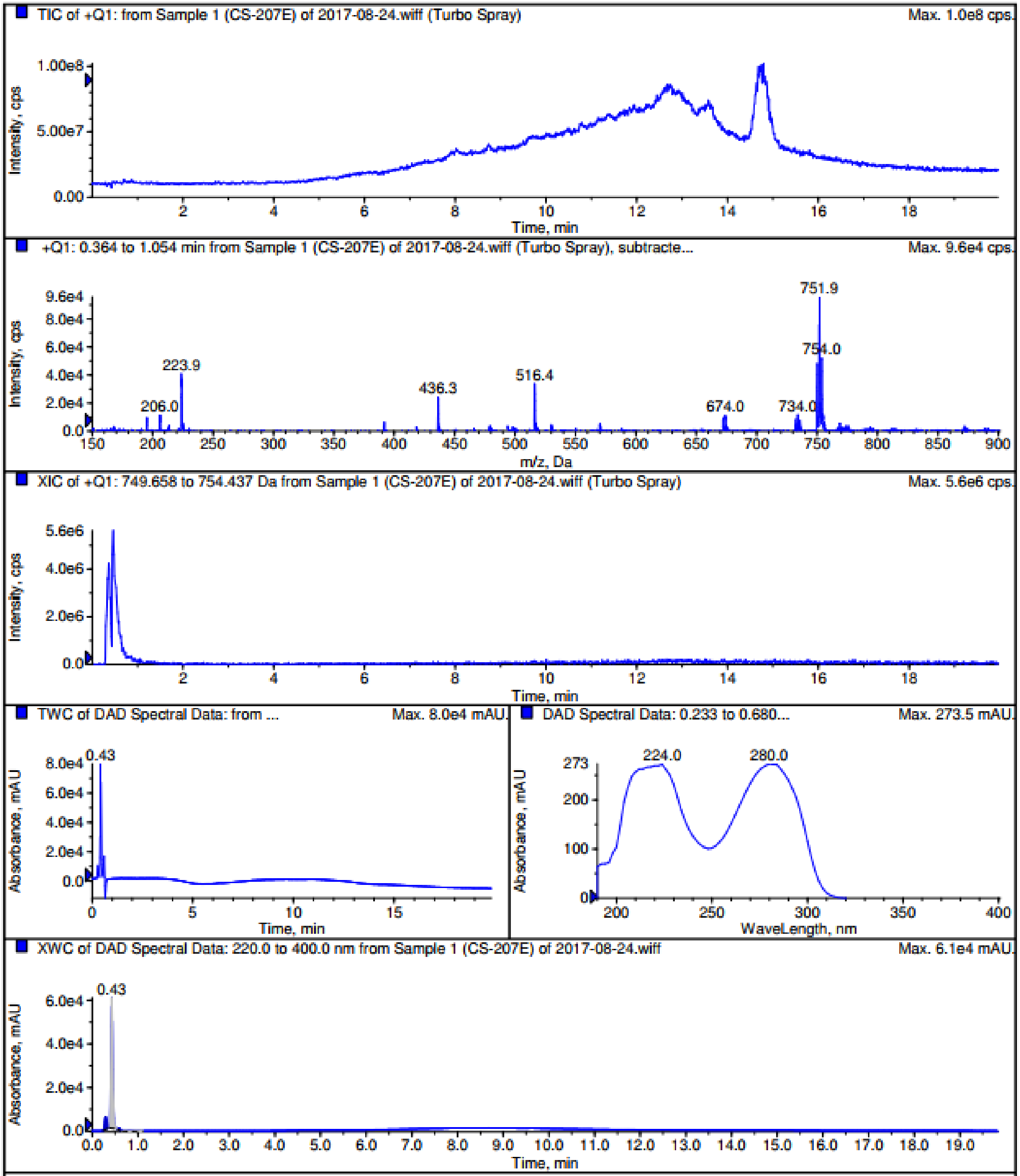

Peak List for "XWC of DAD Spectral Data: 220.0 to 400.0 nm from Sample 1 (CS-207E) of 2017-08-24.wiff"

	Time (min)	Area (mAU x min)	%Area	Height (mAU)	%Height	Width (min)	Baseline Type
1	0.2951	2.1798e4	10.1453	5963.0150	8.8578	0.1400	Base to Base
2	0.4274	1.8298e5	85.1634	6.0032e4	89.1740	0.1800	Base to Base
3	0.5950	2737.5984	1.2741	786.3838	1.1681	0.1000	Base to Base
4	0.7279	7342.1740	3.4172	538.5953	0.8001	0.4600	Base to Base

Figure S4. LC-MS spectrum of compound **31**. The purity of compound **31** was 100% (the peaks correspond to different protonation states)

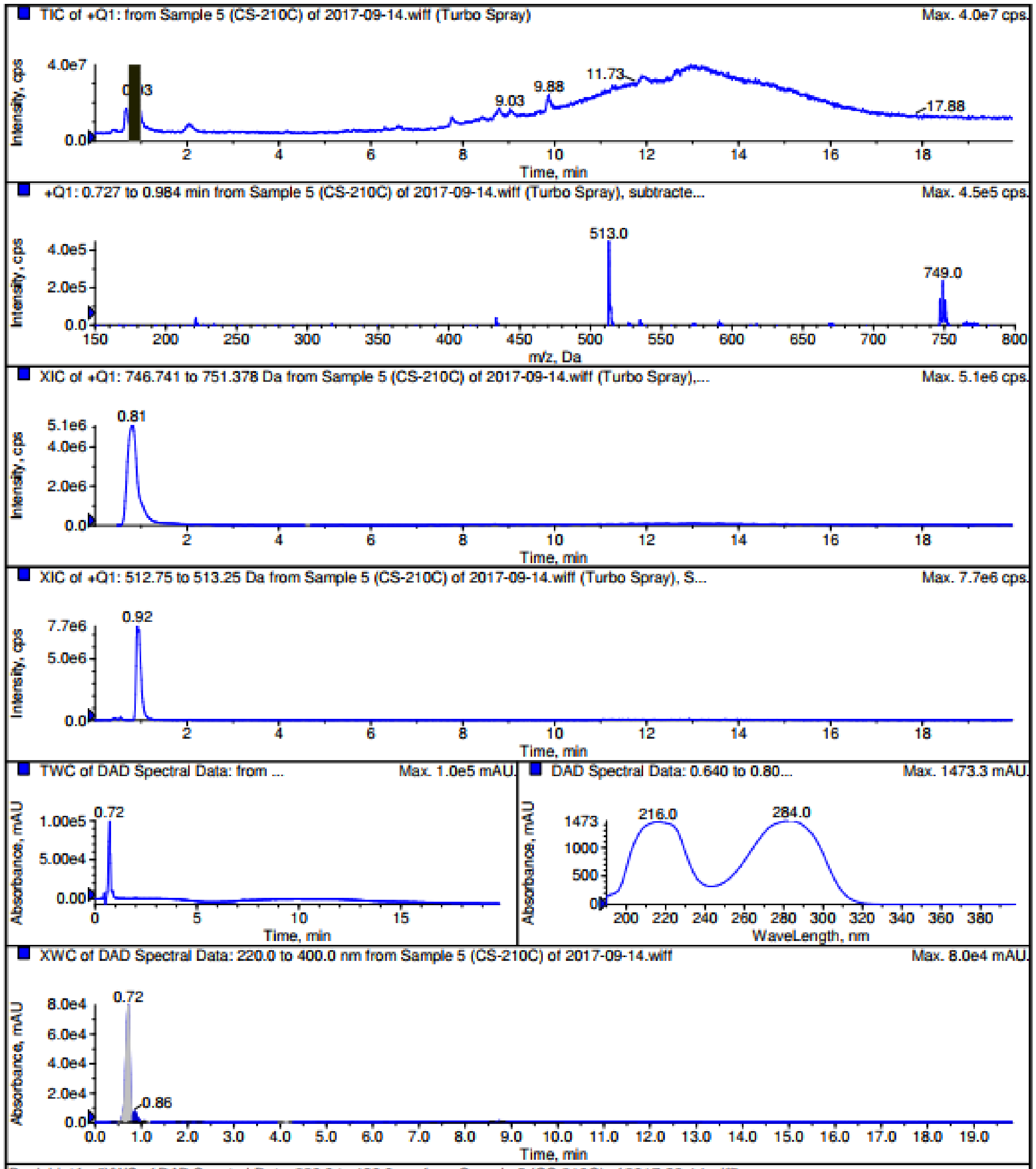

Peak List for "XWC of DAD Spectral Data: 220.0 to 400.0 nm from Sample 5 (CS-210C) of 2017-09-14.wiff"

	Time (min)	Area (mAU x min)	% Area	Height (mAU)	% Height	Width (min)	Baseline Type
1	0.4461	921.2817	0.1776	231.0029	0.2645	0.1333	Valley
2	0.7174	4.7226e5	91.0428	7.9261e4	90.7454	0.3200	Valley
3	0.8563	3.7027e4	7.1380	6763.3335	7.7433	0.2000	Valley
4	1.0682	1292.9810	0.2493	241.6241	0.2766	0.1733	Base to Base
5	1.9777	4326.8272	0.8341	397.6061	0.4552	0.6133	Base to Base
6	4.1083	1147.9435	0.2213	195.2011	0.2235	0.2933	Base to Base
7	8.7338	1747.8257	0.3369	254.5860	0.2915	0.3800	Valley

Figure S5. LC-MS spectrum of compound **33**. The purity of compound **33** was 99% (the peaks correspond to different protonation state)

A

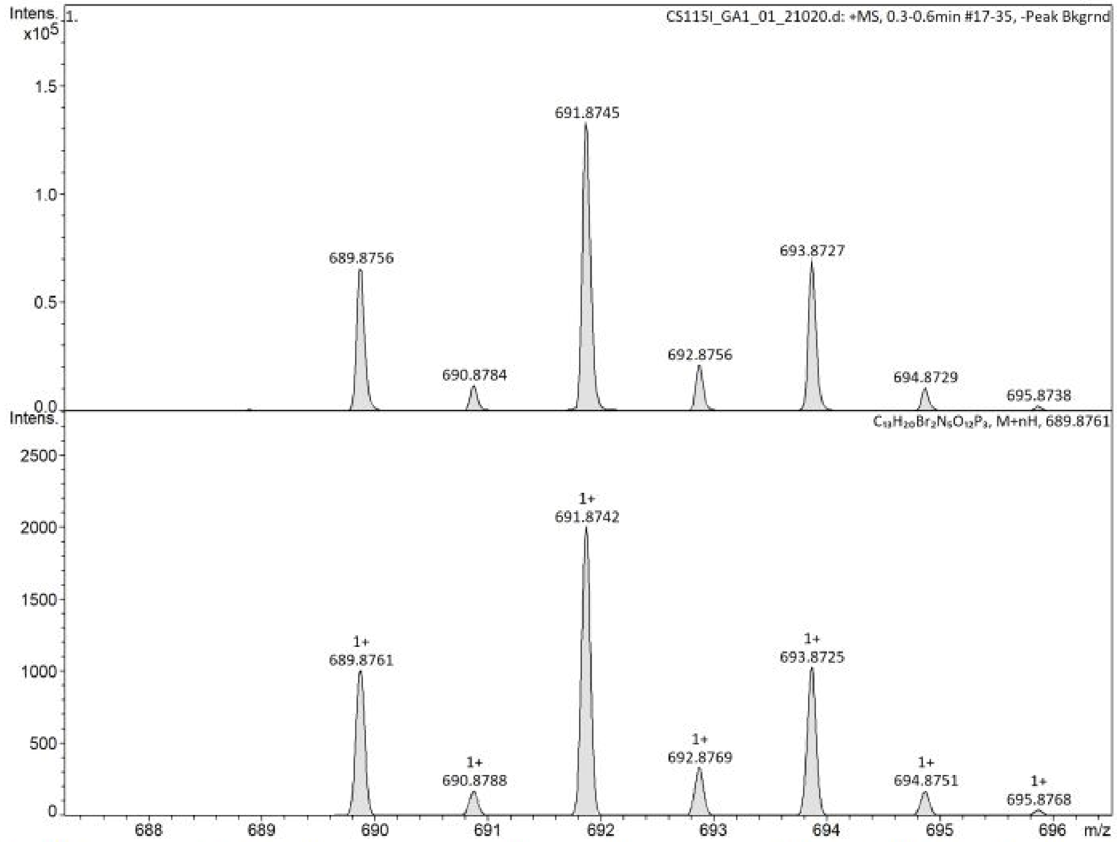

#	RT [min]	Area	Int. Type	I	S/N	Chromatogram	Max. m/z	FWHM [min]	Area Frac. %
1	0.4	1506501.3750	Manual	178260	444.6	BPC +All MS	691.8745		51.3483
2	0.4	608.7229	Manual	112	489832.2	UV Chromatogram, 254 nm	0.0000		97.7812
3	0.4	602.2388	Manual	112	490384.0	UV Chromatogram, 254 nm	0.0000		98.0908
4	2.8	9.3807	Manual	2	10704.9	UV Chromatogram, 254 nm	0.0000		1.5068
5	2.8	11.7220	Manual	2	11895.1	UV Chromatogram, 254 nm	0.0000		1.9092
6	2.8	1427387.7500	Manual	180497	452.0	BPC +All MS	296.1363		48.6517
7	5.6	4.4319	Manual	1	3310.7	UV Chromatogram, 254 nm	0.0000		0.7119

B

Spectrum Data

#	m/z	Res.	S/N	I	I %	FWHM
1	689.8761	7921		1008	50.4	0.0871
2	690.8788	7932		167	8.4	0.0871
3	691.8742	7943		2000	100.0	0.0871
4	692.8769	7955		330	16.5	0.0871
5	693.8725	7966		1029	51.4	0.0871
6	694.8751	7978		168	8.4	0.0871
7	695.8768	7989		37	1.9	0.0871
8	696.8793	8001		5	0.2	0.0871

C

Spectrum Data

#	m/z	Res.	S/N	I	I %	FWHM
1	689.8756	9032	1830.9	65515	49.3	0.0764
2	690.8784	9942	326.7	11690	8.8	0.0695
3	691.8745	8809	3711.9	132825	100.0	0.0785
4	692.8756	9547	585.9	20970	15.8	0.0726
5	693.8727	9517	1926.1	68952	51.9	0.0729
6	694.8729	10794	291.1	10428	7.9	0.0644
7	695.8738	11013	61.5	2207	1.7	0.0632

Figure S6. A. MS data of isotope distribution pattern of compound **24**. **B**. Calculated and **C.** measured isotope fractions of compound **24**.

A

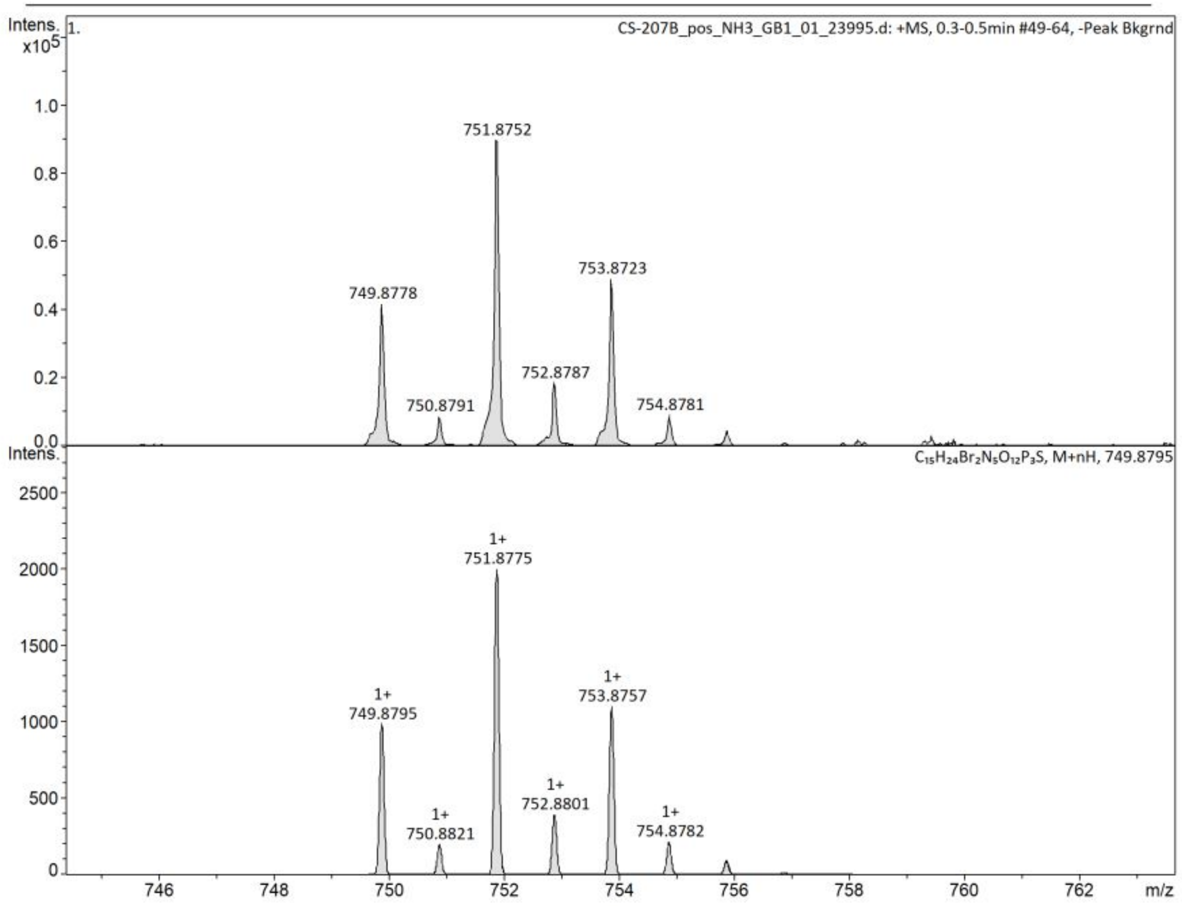

#	RT [min]	Area	Int. Type	I	S/N	Chromatogram	Max. m/z	FWHM [min]	Area Frac. %
1	0.3	866331.6	Manual	111423	28.9	BPC +All MS	751.8752		100.00
2	0.3	2295.2	Manual	645	1742911.1	UV Chromatogram, 220 nm	0.0000		100.00

B

Spectrum Data

#	m/z	Res.	S/N	I	I %	FWHM
1	749.8795	8609		984	49.2	0.0871
2	750.8821	8621		193	9.6	0.0871
3	751.8775	8632		2000	100.0	0.0871
4	752.8801	8644		389	19.4	0.0871
5	753.8757	8655		1101	55.1	0.0871
6	754.8782	8667		210	10.5	0.0871
7	755.8758	8678		87	4.3	0.0871
8	756.8777	8690		14	0.7	0.0871
9	757.8786	8701		2	0.1	0.0871

C

Spectrum Data

#	m/z	Res.	S/N	I	I %	FWHM
1	749.8778	9083	361.9	41563	46.4	0.0826
2	750.8791	10503	74.7	8584	9.6	0.0715
3	751.8752	9123	778.7	89547	100.0	0.0824
4	752.8787	10576	158.0	18186	20.3	0.0712
5	753.8723	9517	424.0	48843	54.5	0.0792
6	754.8781	10302	76.4	8814	9.8	0.0733
7	755.8817	10339	38.1	4402	4.9	0.0731

Figure S7. A. MS data of isotope distribution pattern of compound **31**.**B.** Calculated and **C.** measured isotope fractions of compound **31**.

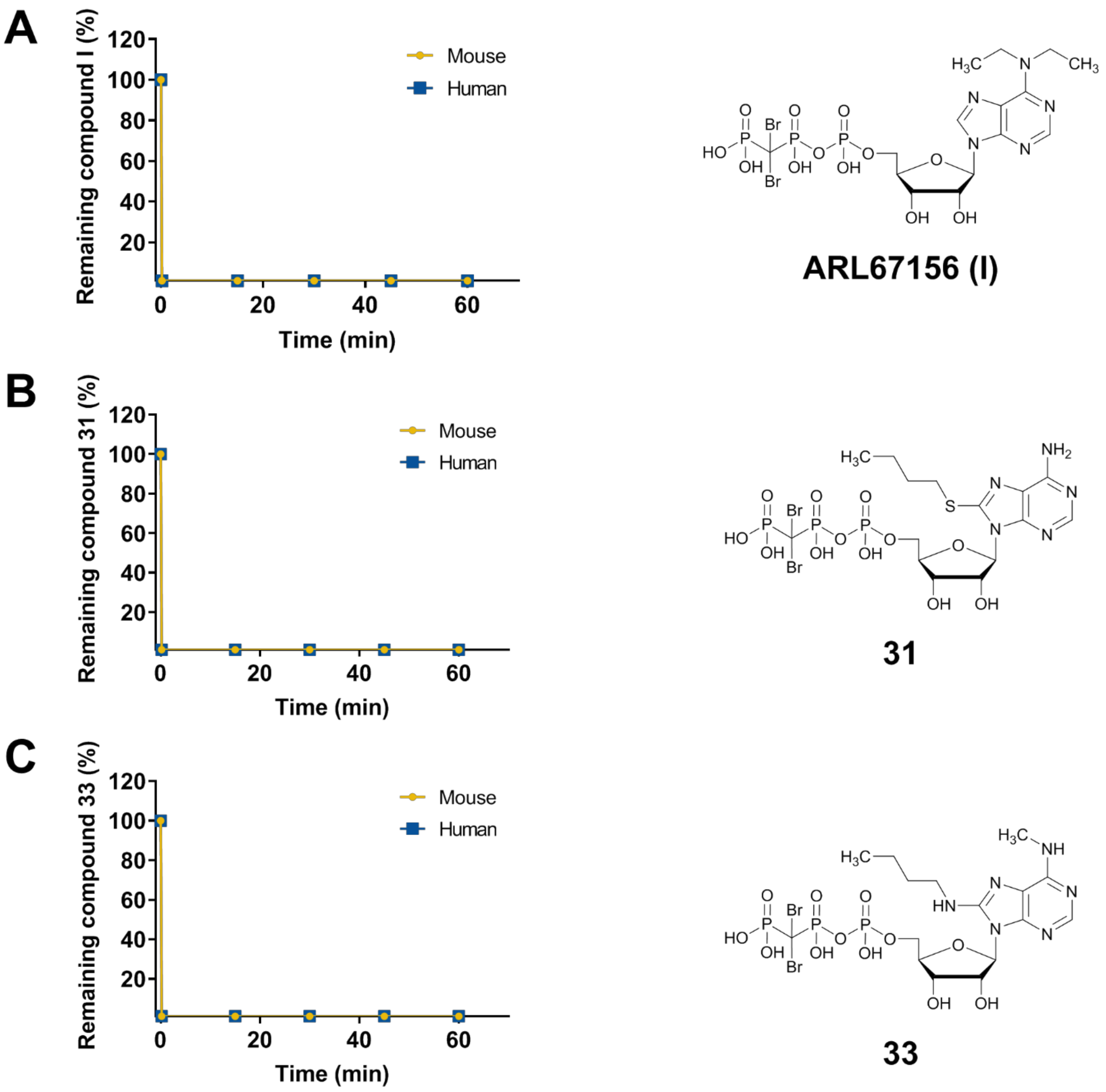

Figure S8. Metabolic stability of ARL67156-derived-CD39-inhibitors. Compounds were tested at a concentration of 1 µM by Pharmacelsus (Saarbrücken, Germany). Effect of human liver microsomes

(0.5 mg/mL, mixed gender, pooled) on the recovery rate of (**A**) ARL67156 (**I**), (**B**) **31** and (**C**) **33** determined by LC/ESI-MS analysis.

A

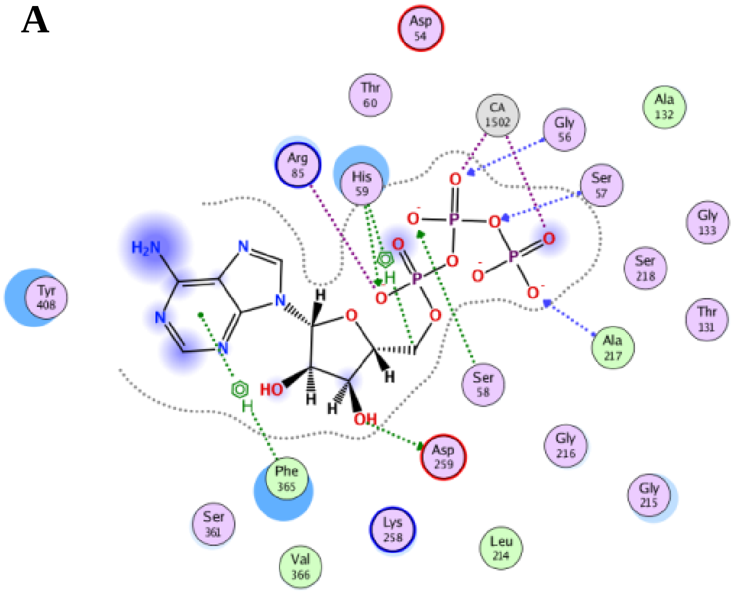

B

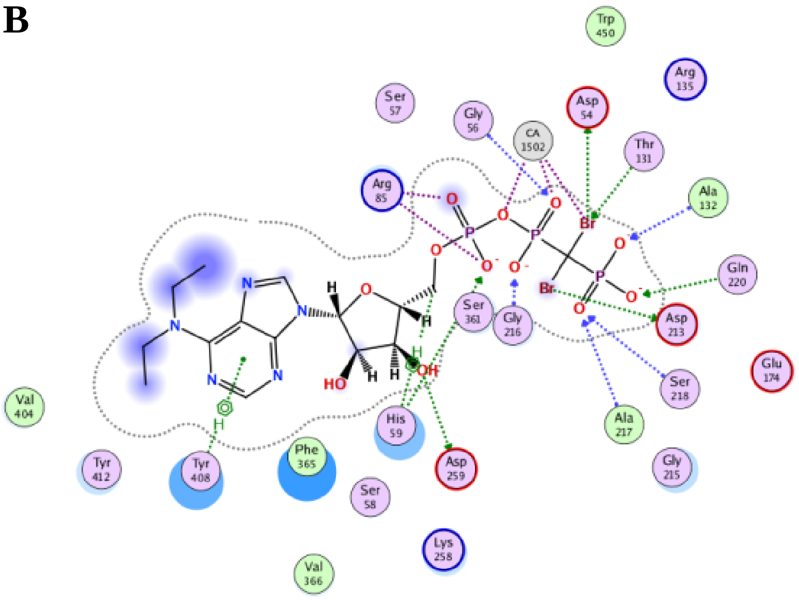

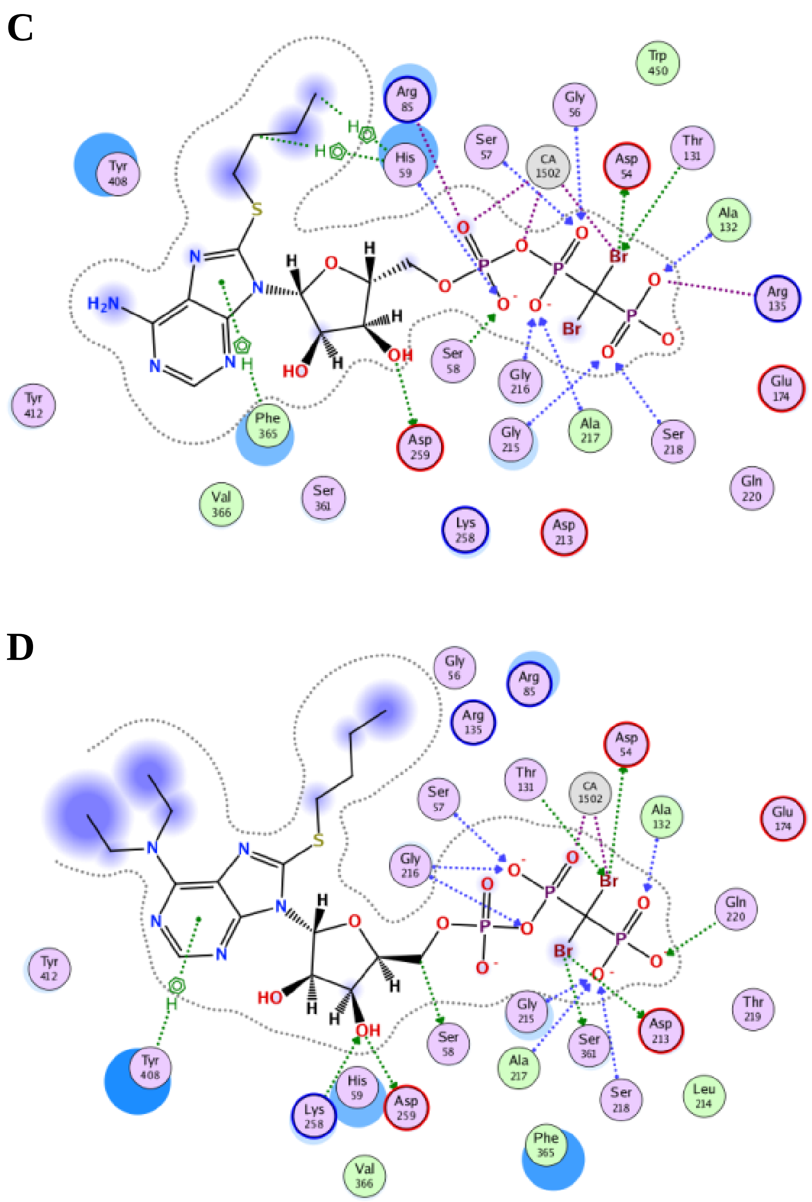

Figure S9. 2D-interaction diagram of nucleotides in the binding site of human CD39. **A**. ATP, **B**. ARL67156 (**I**), **C**. inhibitor **31** and **D**. inhibitor **37**

A

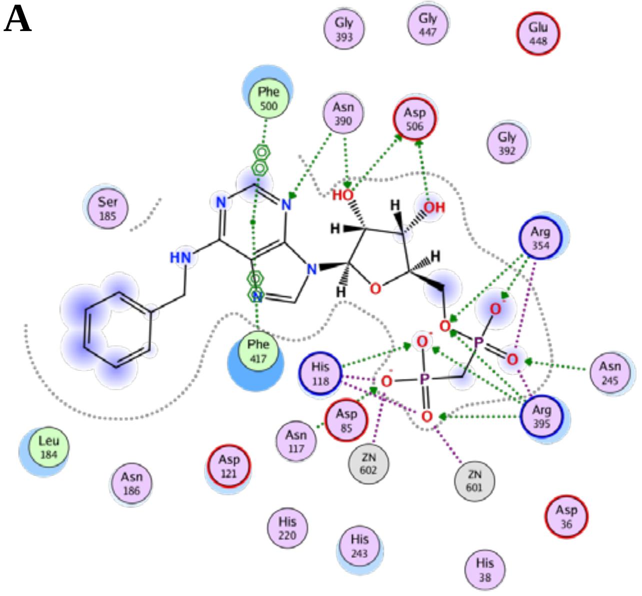
Gly 393
Gly 447
Glu 448
Phe 500
Asn 390
Asp 506
Gly 392
Ser 185
Arg 354
HN
HO
OH
Phe 417
His 118
Asn 245
Asp 85
Arg 395
Leu 184
Asn 117
ZN 602
ZN 601
Asn 186
Asp 121
Asp 36
His 220
His 243
His 38

B

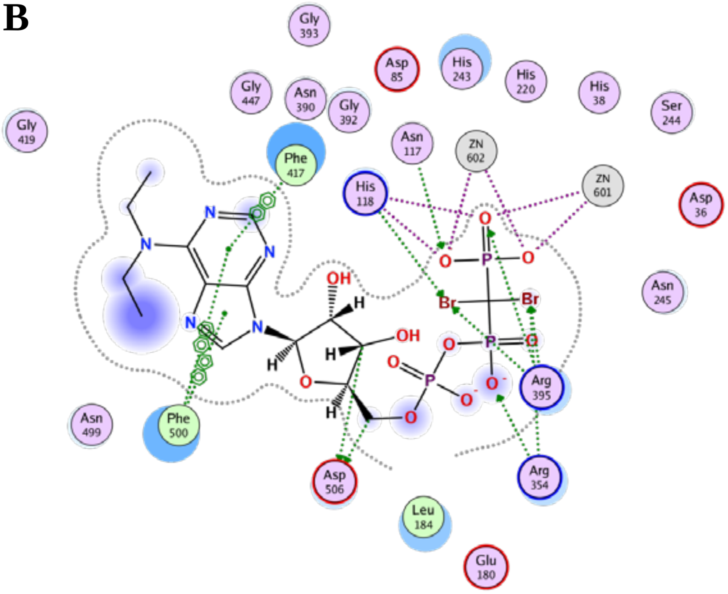
Gly 393
Asp 85
His 243
His 220
His 38
Ser 244
Gly 447
Asn 390
Gly 392
Gly 419
Asn 117
ZN 602
Phe 417
ZN 601
His 118
Asp 36
OH
Br
Asn 245
Arg 395
Asn 499
Phe 500
Arg 354
Asp 506
Leu 184
Glu 180

C

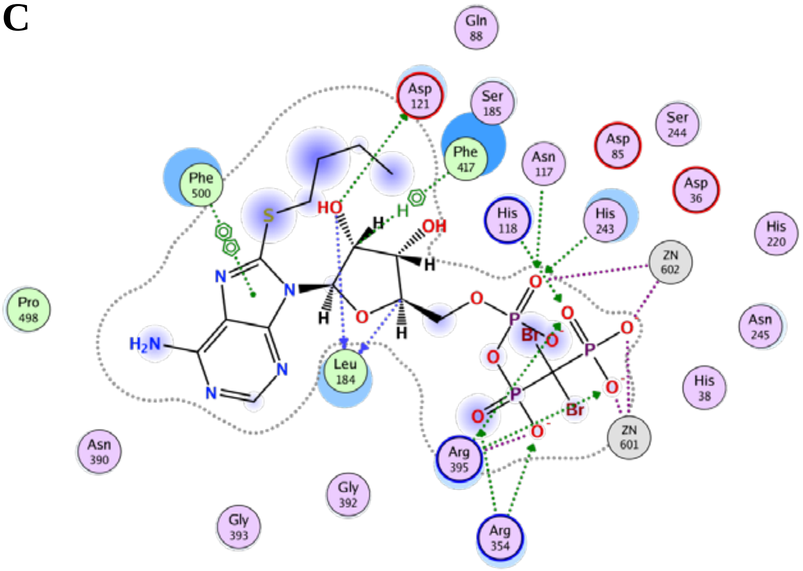

D

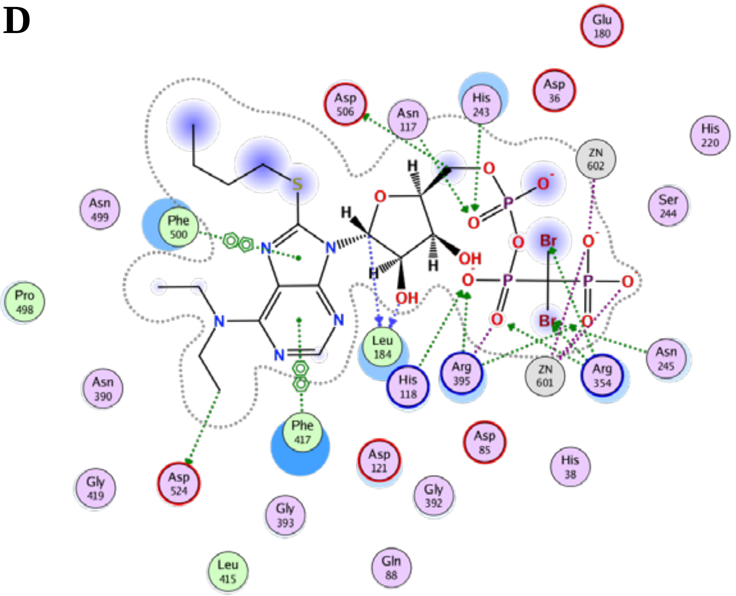

Figure S10. 2D-interaction diagram of nucleotides in the binding site of human CD73. **A**. **PSB-12379**, **B**. ARL67165 (**I**), **C**. inhibitor **31**, and **D**. inhibitor **37**
