# Supplementary figures and images for: Nucleotide Analog ARL67156 as a Lead Structure for the Development of CD39 and Dual CD39/CD73 Ectonucleotidase Inhibitors
Source: Front Pharmacol. 2020 Sep 8;11:1294. doi: 10.3389/fphar.2020.01294 (PMC7508162; doi:10.3389/fphar.2020.01294)

## Slide 1
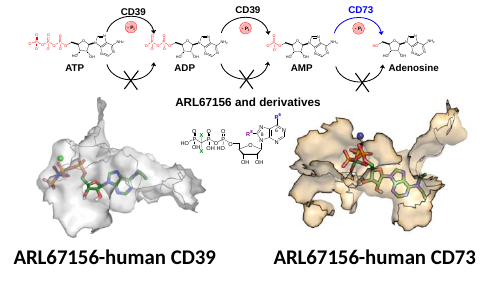

ARL67156-human CD73
ARL67156-human CD39

Supplement: Supplementary file 2 [file Presentation_1.pptx]
